# Dehydration risk is associated with reduced nest attendance and hatching success in a cooperatively breeding bird, the southern pied babbler *Turdoides bicolor*

**DOI:** 10.1093/conphys/coab043

**Published:** 2021-06-16

**Authors:** Amanda R Bourne, Amanda R Ridley, Andrew E McKechnie, Claire N Spottiswoode, Susan J Cunningham

**Affiliations:** 1 FitzPatrick Institute of African Ornithology, DSI-NRF Centre of Excellence, University of Cape Town, Private Bag X3, Rondebosch 7701, South Africa; 2 Centre for Evolutionary Biology, School of Biological Sciences, University of Western Australia, Crawley 6009, Australia; 3 South African Research Chair in Conservation Physiology, South African National Biodiversity Institute, Pretoria 0184, South Africa; 4 DSI-NRF Centre of Excellence at the FitzPatrick Institute, Department of Zoology and Entomology, University of Pretoria, Hatfield 0002, South Africa; 5 Department of Zoology, University of Cambridge, Downing Street, Cambridge CB2 3EJ, UK

**Keywords:** Climate change, cooperative breeding, high temperatures, incubation, parental care, southern pied babbler

## Abstract

High air temperatures have measurable negative impacts on reproduction in wild animal populations, including during incubation in birds. Understanding the mechanisms driving these impacts requires comprehensive knowledge of animal physiology and behaviour under natural conditions. We used a novel combination of a non-invasive doubly labelled water (DLW) technique, nest temperature data and field-based behaviour observations to test effects of temperature, rainfall and group size on physiology and behaviour during incubation in southern pied babblers *Turdoides bicolor*, a cooperatively breeding passerine endemic to the arid savanna regions of southern Africa. The proportion of time that clutches were incubated declined as air temperatures increased, a behavioural pattern traditionally interpreted as a benefit of ambient incubation. However, we show that (i) clutches had a <50% chance of hatching when exposed to daily maximum air temperatures of >35.3°C; (ii) pied babbler groups incubated their nests almost constantly (99% of daylight hours) except on hot days; (iii) operative temperatures in unattended nests frequently exceeded 40.5°C, above which bird embryos are at risk of death; (iv) pied babblers incubating for long periods of time failed to maintain water balance on hot days; and (v) pied babblers from incubating groups lost mass on hot days. These results suggest that pied babblers might leave their nests during hot periods to lower the risk of dehydration associated with prolonged incubation at high operative temperatures. As mean air temperatures increase and extreme heat events become more frequent under climate change, birds will likely incur ever greater thermoregulatory costs of incubation, leading to compromised nest attendance and increased potential for eggs to overheat, with implications for nest success and, ultimately, population persistence.

## Teaser

We have used a novel non-invasive doubly labelled water technique and detailed behaviour observations to show that birds might abandon incubation during hot periods to avoid dehydration as a consequence of incubating at high temperatures; potentially exposing their eggs to the risk of overheating and becoming unviable.

## Introduction

Anthropogenic climate change is driving population declines in birds globally (Iknayan and Beissinger, 2018; Rosenberg *et al.*, 2019; Saino *et al.*, 2011), often via negative impacts on reproduction (Cahill *et al.*, 2013; Cunningham *et al.*, 2013; Stevenson and Bryant, 2000). Many studies have considered the impacts of climate variability and change on birds (Dunn and Møller, 2019; McKechnie, 2019; Pearce-Higgins and Green, 2014). Impacts directly attributable to adverse weather and changing climate regimes include higher risk of mortality (Bourne *et al.*, 2020b; McKechnie and Wolf, 2010; Sharpe *et al.*, 2019), reduced breeding success (Bourne *et al.*, 2020a; Conrey *et al.*, 2016; Cruz-McDonnell and Wolf, 2016; Cunningham *et al.*, 2013; Skagen and Yackel Adams, 2012), compromised body condition and immunocompetence (du Plessis *et al.*, 2012; Edwards *et al.*, 2015; Gardner *et al.*, 2018; Wingfield *et al.*, 2017; Xie *et al.*, 2017), declining populations (Riddell *et al.*, 2019; Saino *et al.*, 2011), range changes (Hockey *et al.*, 2011; Huntley, 2019) and potentially maladaptive behavioural adjustments to foraging (Bladon *et al.*, 2019; Cooper *et al.*, 2019; Cunningham *et al.*, 2015, 2021; Funghi *et al.*, 2019; Pattinson and Smit, 2017), parental care (Bourne *et al.*, 2021; Carroll *et al.*, 2018; Clauser and McRae, 2017; van de Ven, 2017; Wiley and Ridley, 2016) and migration (Dunn *et al.*, 2010; Samplonius *et al.*, 2018).

Hatching failure in birds is particularly common during hot weather (Bourne *et al.*, 2020a; Clauser and McRae, 2017; Wada *et al.*, 2015) and droughts (Conrey *et al.*, 2016), both of which are becoming more frequent under climate warming (Ripple *et al.*, 2019). Eggs of most birds are incubated at temperatures averaging ~ 35.5°C (Drent, 1975) and egg temperatures higher than this are likely to be lethal (Walsberg and Voss-Roberts, 1983; Webb, 1987).

Incubation is energetically costly in temperate environments where eggs need to be kept warm (Ardia *et al.*, 2010; Nord *et al.*, 2010; Nord and Cooper, 2020), but also extremely challenging in warm environments (Amat and Masero, 2004; Coe *et al.*, 2015; Nwaogu *et al.*, 2017), where incubating birds must prevent eggs from overheating (Carroll *et al.*, 2015a; Grant, 1982; McDonald and Schwanz, 2018) while also thermoregulating themselves (DuRant *et al.*, 2019; McKechnie, 2019; O’Connor *et al.*, 2018). Behaviourally, birds initially respond to high temperatures by increasing incubation constancy (AlRashidi *et al.*, 2011; Cones, 2017; Conway and Martin, 2000; Mortensen and Reed, 2018; Mougeot *et al.*, 2014) or engaging in shading behaviour (Brown and Downs, 2003; Clauser and McRae, 2017; Downs and Ward, 1997; Grant, 1982) in order to regulate nest temperatures. Physiologically, the capacity of small endotherms such as birds to tolerate heat exposure is governed by their ability to dissipate heat (McKechnie and Wolf, 2019). In free-living birds, high air temperatures are associated with lower metabolic rates (Bourne *et al.*, 2019; Smit and McKechnie, 2015), dehydration (Bourne, 2020; Sharpe *et al.*, 2019), higher glucocorticid levels (Moagi *et al.*, 2021), impaired cognitive function (Soravia *et al.*, 2021) and even death (Conradie *et al.*, 2020; McKechnie *et al.*, 2012). As incubating birds reach limits in their ability to tolerate high temperatures over long periods, they undertake more frequent or longer incubation recesses (Bourne, 2020; Clauser and McRae, 2017) and may ultimately abandon their nests (Clauser and McRae, 2017; Sharpe *et al.*, 2019). Understanding the behavioural and physiological mechanisms driving hatching failure at high temperatures *in situ* in wild populations is critical to our ability to predict species-specific responses to climate change (Conradie *et al.*, 2019; Stillman, 2019).

Here we present the first study of avian reproduction combining both direct observations of incubation behaviour under natural conditions and non-invasive physiological measurements from the same individuals at the same time. We investigate climate effects on the behaviour and physiology of incubating adults in southern pied babblers *Turdoides bicolor* (hereafter ‘pied babblers’), a cooperatively breeding bird. Pied babblers live in groups ranging in size from 3 to 12 adults (Ridley 2016). Adults are defined as individuals aged ≥12 months (Raihani and Ridley, 2007a) and groups consist of a dominant pair and one or more subordinate adults of either sex (Nelson-Flower *et al.*, 2011). Air temperatures between 35°C and 38°C are known to correlate with negative impacts in pied babblers. At air temperatures above ~35.5°C, pied babbler eggs are half as likely to hatch (Bourne *et al.*, 2020a), adult birds typically do not gain enough body mass during the day to offset overnight mass loss (du Plessis *et al.*, 2012) and provisioning to nestlings declines (Wiley and Ridley, 2016). No breeding attempts produce surviving young at air temperatures exceeding 38°C (Bourne *et al.*, 2020a). High average air temperatures during summer are associated with dramatically reduced survival probabilities in adult pied babblers, particularly when these occur in combination with drought (Bourne *et al.*, 2020b; Ridley *et al.*, 2021). Additionally, faecal glucocorticoid levels are elevated in pied babblers at air temperatures above 38°C (Moagi *et al.*, 2021), indicative of an acute physiological response to high temperatures.

Cooperative species may respond differently to environmental variability compared to pair-breeding or solitary species, because reproductive investment and nest outcomes can be influenced by the presence of helpers (van de Ven *et al.*, 2020; Wiley and Ridley, 2016), and so we also considered the influence of the number of adults present in each group and checked for interactions between group size and climate variables (Rubenstein and Lovette, 2007). We hypothesized that high T_air_ would reduce hatching rates via reduced nest attendance as a result of thermoregulatory costs on incubating adults. This would increase risk of lethal heat exposure for developing embryos. We addressed this hypothesis by testing predictions related to (i) nest outcomes (lower probability of hatching at high T_air_); (ii) incubation behaviour (reduction in the proportion of time nests are attended at high T_air_); (iii) the temperatures reached in unattended nests at high T_air_ (exceeding lethal limits for avian embryos, explaining why hot nests are less likely to hatch); and (iv) physiological costs of incubation for adults (higher costs of incubation at higher T_air_ evident in patterns of energy expenditure, water balance and body mass maintenance). We tested part of the latter prediction using a novel, non-invasive DLW technique (Anava *et al.*, 2000; Bourne *et al.*, 2019). We further expected that larger group sizes would be associated with reduced costs of incubation at higher T_air_ and improved nest outcomes in our semi-arid study system.

## Materials and Methods

Unless otherwise indicated, summary statistics are presented as mean ± one standard deviation.

### Study site and system

Fieldwork took place at the 33km^2^ Kuruman River Reserve (26°58’S, 21°49′E) in the southern African Kalahari. Mean summer daily maximum temperatures in the region averaged 34.7 ± 9.7°C and mean annual precipitation averaged 186 ± 88 mm (1995–2015; van de Ven, McKechnie and Cunningham 2019). Rainfall has been declining and high temperature extremes increasing in both frequency and severity over the past 20 years (Bourne *et al.*, 2020a; Kruger and Sekele, 2013; van Wilgen *et al.*, 2016).

Pied babblers are medium-sized (60–90 g), cooperatively breeding passerines that live in groups ranging in size from 3 to 15 adults (Raihani and Ridley, 2007b) and are endemic to the Kalahari (Ridley, 2016). Resident, territorial groups consist of a single breeding pair (one dominant male and female) with subordinate helpers of both sexes (Nelson-Flower *et al.*, 2011) and can be reliably located by visits to each territory (Ridley, 2016). Individuals in the study population are habituated to observation by humans at distances of 1–5 m (Ridley and Raihani, 2007) and are individually identifiable by a unique combination of metal and colour leg rings.

Pied babblers build open cup nests, usually in camelthorn *Vachellia erioloba* trees and usually breed during spring and summer (Bourne *et al.*, 2020a; Ridley, 2016). During each breeding attempt, a clutch of ~3 eggs is laid and incubated for 13–15 days (Bourne *et al.*, 2020a; Ridley and Raihani, 2008). While only the dominant female incubates overnight (Ridley, 2016), during the day all adult group members (individuals, >1 year old), including subordinates, take turns to incubate and the nest is rarely left unattended for more than a few minutes at a time (Ridley and Raihani, 2007; Ridley and van den Heuvel, 2012). Pied babblers will drink water when it is available, but can obtain all of their water from their food, and at least two of the groups in the study population do not have access to water in their territories.

### Data collection

Data were collected during each austral summer breeding season between September 2016 and February 2019 (three breeding seasons). We recorded air temperature (°C), solar radiation (W·m^−2^), wind speed (m·s^−1^), relative humidity (%) and rainfall (mm) using an on-site weather station (Vantage Pro2, Davis Instruments, Hayward, USA; factory calibration with accuracy = 0.3°C). For our analyses, we caculated daily maximum air temperature (T_max_), daily maximum solar radiation (Sol_max_) and daily maximum wind speed (Wind_max_) for each observation day and total rainfall in the two months prior to each observation day (mm). We calculated absolute humidity (g·m^−3^) for each pair of air temperature and relative humidity values (Campbell and Norman, 1988) and calculated the absolute humidity value coinciding with T_max_ (AbsHum_Tmax_). For analyses of nest outcomes, we additionally calculated average T_max,_ Sol_max_, Wind_max_ and AbsHum_Tmax_ between initiation of incubation and hatching (MeanT_maxInc,_ MeanSol_maxInc_, MeanWind_maxInc_ and MeanAbsHum_TmaxInc_). We recorded group size (number of adults) during each breeding attempt in each group. T_max_ ranged from 20.7°C to 40.8°C (mean = 34.1 ± 4.5), Sol_max_ from 186 to 1383 W·m^−2^ (mean = 999 ± 150), Wind_max_ from 0 to 8.9 m·s^−1^ (mean = 4.2 ± 1.4), AbsHum_Tmax_ from 0.8 to 14.4 g·m^−3^ (mean = 5.2 ± 3), rainfall from 0.2 to 140.2 mm (median = 15) and group size from 3 to 6 adults (mean = 4 ± 1).

#### Nest outcomes

Monitoring of nest outcomes (99 breeding attempts by 23 pied babbler groups, with mean = 4, range of 1–10 nests per group, over 3 breeding seasons) followed Ridley and van den Heuvel (2012). Breeding attempts were defined as discrete clutches laid and incubated. Nests were located by observing nest building during weekly monitoring visits. Once located, the nests were checked approximately every 2 days to identify incubation start and hatch dates: nests were categorized as hatched when adult group members were observed carrying food items to the nest and as failed when nests were left unattended for longer than 90 minutes on two consecutive monitoring visits or the group was observed building a new nest. Incubation starts when all eggs are laid and the dominant female begins to incubate overnight.

#### Incubation behaviour

Incubation bout and recess data were collected by waiting near the nest at dawn, observing the first bird to replace the dominant female in the morning (05 h00–06 h48) and remaining with the group all day until 19 h00 (46 observation days at 35 nests). Observations were collected once during the incubation period for most nests and on two or more days (up to a maximum of 4 days) for 8 nests. We recorded the start and end time of each incubation bout and the duration of any time periods during which the nest was left unattended (recesses). These data were used to calculate the proportion of time per day that clutches were incubated (sum of all incubation bout durations per day/total observation time). Both members of the dominant pair incubated on every observation day, with the help of at least one subordinate group member on most (91%) days. In over 90% of cases, the incubating bird did not leave the nest until it was replaced by another, therefore making it unlikely that many incubation recesses were missed.

#### Nest temperatures

To quantify variation in the thermal properties of pied babbler nests, we measured operative temperature [T_e_: a measure of thermal load experienced by the bird (Bakken *et al.*, 1985)] using black bulb thermometers (Bakken *et al.*, 1985, 2001; Carroll *et al.*, 2015; Cunningham *et al.*, 2015; Howell and Bartholomew, 1961; Pattinson and Smit, 2017) placed in 23 nests within 5 days of fledge/fail (Griffith *et al.*, 2016), recording constantly for ~2 weeks (12.5 ± 3 days; range, 9–20 days; *n* = 21 872 records of daytime T_e_ in total). Weather conditions were not significantly different between the active next period and the time periods during which T_e_ was recorded in the nests (paired *t*-tests, all *P* > 0.05; see Fig. S1, Table S1). Black bulb thermometers comprised two copper half spheres (which approximates pied babbler thoracic cavity dimensions; diameter, 42 mm; thickness, 0.8 mm), sealed together using cryanoacrylate adhesive, painted matte black (Carroll *et al.*, 2015a; van de Ven *et al.*, 2019) and containing internally mounted temperature loggers (Thermocron iButton, DS1923, Maxim, Sunnyvale, CA, USA; resolution, 0.0625°C) logging at 10-minute intervals (Cunningham *et al.*, 2015; van de Ven *et al.*, 2019) synchronized with T_air_ records from the onsite weather station. The iButton loggers were calibrated in a circulating water bath against a factory-calibrated NiCr-NiAl thermocouple (Thermocouple HH21A, Omega Engineering, Stamford, USA; van de Ven, 2017).

Black bulb thermometers do not provide a complete representation of thermal conditions experienced by incubating pied babblers because they mimic neither feather arrangement nor colour (Carroll *et al.*, 2017) and do not account for humidity or evaporative heat loss (Bakken *et al.*, 1985). Nonetheless, they provide a relative measure of differences in temperature across nest microsites, which cannot be quantified by measuring T_air_ alone (Cunningham *et al.*, 2015). We also acknowledge that using T_air_ as a predictor variable for babbler behaviour, physiology and nest outcomes (below), instead of estimated T_e_ experienced by incubating adults, imposes limitations on the interpretation of our data. We opted for this approach for two reasons. First, accurate measurements of species-specific T_e_ experienced by incubating adults would require the use of taxidermic mounts calibrated against measurements of evaporative water loss and metabolic rate in babblers exposed to a range of wind speeds and solar irradiance levels under laboratory conditions (Walsberg and Wolf, 1996), an undertaking beyond the scope of this study. Second, although T_air_ is a crude index of pied babbler’s thermal environments, this approach ensures our data are comparable to those reported in previous studies evaluating the fitness costs of periods of hot weather (Cooper *et al.*, 2019; du Plessis *et al.*, 2012; Edwards *et al.*, 2015; Sharpe *et al.*, 2019; van de Ven *et al.*, 2019; van de Ven *et al.*, 2020) and allows our data to be useful in the context of models of future climate, which inevitably use T_air_ rather than species- or site-specific T_e_. Synchronized recording intervals enabled comparisons between nest-specific T_e_ and simultaneously occurring T_air_. However, the differences between nest-site T_e_ recorded by the black bulb thermometers and T_air_ recorded by the weather station reiterate that the latter is a crude approximation of the thermal environments experienced by pied babblers. Future studies investigating hot weather effects could benefit from including measures of T_e_ instead of or as well as T_air_ as a predictor.

#### Energy expenditure and water balance

During observation days on which incubation bout and recess data were recorded, we also obtained detailed physiology [daily energy expenditure (DEE) and water balance] and behaviour (incubation effort) data for a subset of adult birds from the incubating groups (up to four individuals per observation day; mean = 1.6 ± 0.9; *n* = 70 individuals in total). We obtained physiology data from individuals across a range of T_max_ values [35 measured on ‘hot’ days, T_max_ ≥ 35.5°C, identified as a critical temperature threshold in pied babblers (Bourne *et al.*, 2020a; du Plessis *et al.*, 2012; Wiley and Ridley, 2016); 35 on ‘cool’ days, T_max_ < 35.5°C] and group sizes (3–6 adults), as well as both sexes (38 females, 31 males, 1 unknown sex) and ranks (40 dominant birds, 30 subordinate birds). Data on DEE (kJ g^−1^ day^−1^) and water balance were collected using a non-invasive DLW technique (Anava *et al.*, 2000; Scantlebury *et al.*, 2014), recently validated and described in detail for pied babblers (Bourne *et al.*, 2019).

In brief, selected individuals were dosed with ~50 μL of DLW—a non-toxic isotopic solution enriched with oxygen-18 (measured as δ^18^O) and deuterium (measured as δ^2^H)—injected into beetle larvae *Zophobias morio* and fed to the birds between 06 h00 and 09 h00 on the observation day. Body water samples were then obtained during all daylight hours over a 24-hour observation period by collecting droppings from dosed individuals as they were excreted naturally onto the ground. Water samples were extracted from droppings by cryogenic distillation, using a technique adapted from Priyadarshini *et al.* (2016) and analysed in a PAL autosampler and DLT-100 liquid water isotope analyser (Los Gatos Research, Mountain View, CA, USA) following the procedures described by Smit and McKechnie (2015) and Bourne *et al.* (2019). We calculated CO_2_ production (*r*CO_2_) from the body water pool and the rate of decline of the natural log of the ratio of δ^18^O/δ^2^H (Nagy and Costa, 1980; Speakman, 1997). We used Speakman’s (1997, Equation 17.7; see Equation (1) below) for calculations of *r*CO_2_ in mol d^−1^ because empirical testing has shown this equation to be the most accurate (Visser *et al.*, 2000) and based on the most realistic assumptions of fractionation during evaporation (Butler *et al.*, 2004; Speakman and Hambly, 2016):

(1) \begin{equation*} r{CO}_2=\left(\frac{N}{2.078}\right)\left({k}_O-{k}_H\right)-0.0062\ast {k}_H\ast N, \end{equation*}

where *N* is moles of body water and values of *k* represent turnover of an isotope identified by the subscript. The divisor of *N* (2.078) accounts for the fact that each molecule of CO_2_ expired removes two molecules of oxygen from the pool and, with the inclusion of the last term (0.0062 • *k*_H_ • *N*), reflects a correction for fractionation. We calculated *k*_H_ in the final term of Equation (1) based on change in ln(*δ*^2^H) between maximally enriched samples collected at early time points and final samples, where *t* is time (in days) elapsed between early and final samples:

(2) \begin{equation*} {k}_H=\frac{\ln \left[{\delta}^2{H}_{1-\mathit{\max}}\right]-\ln \left[{\delta}^2{H}_f\right]}{t} \end{equation*}

Values of $\Big({k}_O-{k}_H\Big)$ can be calculated from the rate of decline of ln($\frac{\delta^{18}{O}_i}{\delta^2{H}_i}$) (Nagy and Costa, 1980; Speakman, 1997):

(3) \begin{equation*} \left({k}_O-{k}_H\right)=\left(\mathit{\ln}\left[\frac{\delta^{18}{O}_i}{\delta^2{H}_i}\right]-\mathit{\ln}\left[\frac{\delta^{18}{O}_f}{\delta^2{H}_f}\right]\right)\ast \left(\frac{1}{t}\right), \end{equation*}

where *δ*^18^O_i_ and *δ*^2^H_i_ are the initial *δ*^18^O and *δ*^2^H values in faeces or blood and *δ*^18^O_f_ and *δ*^2^H_f_ are the final *δ*^18^O and *δ*^2^H values. *r*CO_2_ was converted from mol d^−1^ to L d^−1^ using the conversion factor 22.4 l of ideal gas per mol at standard temperature and pressure, and L CO_2_ d^−1^ was converted to kJ d^−1^ using the relationship 27.42 kJ/l CO_2_ for an insectivorous bird (Gessaman and Nagy, 1988) and used to estimate DEE (otherwise known as field metabolic rate, in kJ g^−1^ d^−1^).

Water balance was calculated by dividing water influx by water efflux, where values >1 indicate positive water balance (a hydrated status) and values <1 indicate negative water balance (a dehydrated status). We used Nagy and Costa’s (1980) Equation 4 (see Equation (4) below) and Equation 6 (see Equation (5) below) to calculate water efflux and water influx (ml H_2_O kg^−1^d^−1^), respectively:

(4) \begin{align*}& \frac{ml{H}_2O\ efflux}{kg\ day}\nonumber\\&=\frac{\mathrm{2,000}\left({W}_2-{W}_1\right)\log \left[\left({H}_1\times {W}_1\right)\div \left({H}_2\times {W}_2\right)\right]}{\left({M}_1+{M}_2\right)\left[1-\left({W}_2\div {W}_1\right)\right]t}, \end{align*}

(5) \begin{equation*} \frac{ml{H}_2O\ influx}{kg\ day}=\frac{ml{H}_2O\ efflux}{kg\ day}+\frac{\mathrm{2,000}\left({W}_2-{W}_1\right)}{t\left({M}_1+{M}_2\right)}, \end{equation*}

where the subscripts 1 and 2 represent initial and final values respectively, *H* = measured deuterium enrichment levels, *M* = body mass in grams, *W* = the body water pool and *t* = time in days between initial and final sampling of deuterium enrichment levels. The body water pool was estimated as 69.3% of body mass, based on measured total body water in 6 pied babblers at a nearby site in a similar habitat (Bourne *et al.*, 2019). If mean body water were 3% lower or higher in the individuals in this study than the average we used, then mean DEE would have been about 3% lower or higher than we calculated (Bourne *et al.*, 2019). This is an acceptable consequence that follows standard practice in the single-sample DLW method, where percentage body water by mass is typically measured in a sample of other individuals (Niizuma and Shirai, 2015; Speakman, 1997) and applied as a constant to a study population.

In order to estimate the number of extra prey items pied babblers would need to eat to make up any water deficit and maintain water balance at high temperatures, we converted DEE to metabolic water production (g d^−1^, Equation (6)) and averaged these values for extreme temperatures in the dataset (≥39°C, *n* = 6; <26°C, *n* = 4):

(6) \begin{equation*} Metabolic\ Water\ Production=\left(\frac{DEE\ast x\ast M}{1000}\right), \end{equation*}

where *x* is the average value of 27 mg kJ-1 for the rate of metabolic water production from fuel oxidation (Schmidt-Nielson, 1990), based on typical macronutrient composition of mealworm *Tenebrio molitor* larvae (Liu *et al.*, 2020; Siemianowska *et al.*, 2013), and *M* is the body mass of the bird in grams. Beetle larvae are a common prey consumed by pied babblers and, in terms of preformed water, mealworms are similar to species regularly consumed by pied babblers. Mealworms weigh ~0.2 g (Raihani and Ridley, 2007a) and are 56% water (Siemianowska *et al.*, 2013).

Because continuous attention is required to collect faecal samples from wild, free-living birds, it was generally only possible to collect detailed behaviour data from one bird per observation day. To identify the proportion of time adult birds dosed with DLW allocated to incubation, we used data collected during ~4 × 20-minute continuous time-activity focal behaviour observations (‘focals’; Altmann, 1974) within each of 6 focal sessions per day (mean = 23 focals per bird per day; range, 15–27; *n* = 48 focal days; data were collected from 2 birds on the same day on 5 occasions, i.e. 10 of the focal days). Focal sessions lasted 2 hours each, with the first starting at 07 h00 and the last at 17 h00, and the data were captured on an Android smartphone (Mobicel Trendy), using Prim8 software (McDonald and Johnson, 2014) in which the duration of each observed behaviour is recorded to the nearest second.

#### Body mass

To determine effects of weather and social factors on body mass maintenance of adults from groups incubating clutches, body mass data were collected from as many adult group members as possible on observation days (mean = 2.6 ± 1.4 measurements per observation day; range, 1–5). These data were obtained by enticing individuals to stand on a top pan balance in exchange for a small food reward (Ridley, 2016), and were collected at dawn on the morning of each observation day (Mass_1_) and again at dawn the following morning (Mass_2_). Body mass change (∆M_b_) was calculated in grams as Mass_2_ − Mass_1_ [*n* = 129; pied babblers are size monomorphic (Bourne *et al.*, 2018; Ridley, 2016) and individuals in the study had similar starting weights (mean = 75.8 g, coefficient of variation = 0.07; Table S2), so using a relative measure (Mass_2_ − Mass_1_/Mass_1_) did not change interpretation of the models].

### Statistical analyses

Statistical analyses were conducted in the R statistical environment, v 3.6.0 (R Core Team, 2017), primarily using mixed-effects models in the package *lme4* (Bates *et al.*, 2015). Model checking and model selection followed Harrison *et al.* (2018). All continuous explanatory variables were centred by subtracting the mean and scaled by dividing by the standard deviation. Additive models were built from significant terms tested in univariate models. All explanatory variables were tested for correlation with one another and correlated variables (VIF > 2) were not included in the same additive models. Akaike’s information criterion corrected for small sample size (AICc) with maximum likelihood estimation was used to determine which models best explained patterns of variation in the data; model estimates with confidence intervals that did not intersect zero were considered to explain significant patterns within our data, and model fits were evaluated using Normal Q-Q plots, histograms of residuals and dispersion parameters as appropriate (Bates *et al.*, 2015). Rainfall in the two months prior to initiation of incubation was correlated with breeding season (*F_2,67_* = 10.994; *P* < 0.001). We chose the categorical variable ‘breeding season’ for all analyses due to the fact that high rainfall only occurred during one breeding season (2016/2017). Quadratic terms for continuous predictors were included when there was no significant linear effect and visualization of the data suggested a non-linear relationship. Where several models were within 2 AICc of the top model, top model sets were averaged (Burnham and Anderson, 2002; Symonds and Moussalli, 2011) using the package *MuMin* (Barton, 2015) and model-averaged coefficients were presented. Sensitivity power analysis (Champely *et al.*, 2018; Greenland *et al.*, 2016) suggested sufficient sample size to detect all main effects, but limited power to detect interactions given our data (Table S3).

To determine which variables predicted (i) nest outcomes (hatched = 1, failed = 0) and (ii) the overall proportion of time clutches were incubated per day (time incubated/time observed), we used generalized linear mixed-effects models with binomial error structure and logit link function. We considered the influence of breeding season, weather [for (i) MeanT_maxInc,_ MeanSol_maxInc_, MeanWind_maxInc_ and MeanAbsHum_TmaxInc_; for (ii) T_max,_ Sol_max_, Wind_max_ and AbsHum_Tmax_ on observation day], group size, group size^2 and the interactions between breeding season and group size and T_max_ and group size. To account for repeated measures and thus for nonindependence of data, we included nest identity as a random factor. For (ii), we further included an observation level random factor to address overdispersion in the data (Harrison, 2014). The inclusion of group identity as a random term in addition to nest identity resulted in unstable models and, of the two random terms, nest identity explained the greatest proportion of variation while avoiding destabilizing the models (Grueber *et al.*, 2011; Harrison *et al.*, 2018).

To determine which variables predicted DEE (*n* = 68) and water balance (*n* = 69), we used maximum likelihood linear mixed-effects models (LMMs) to test the following predictors: breeding season, T_max_, Sol_max_, Wind_max_, AbsHum_Tmax_, group size, sex, rank and the interactions between breeding season and group size and T_max_ and group size. For a subset of individuals for which we collected both behaviour and physiology data from the same birds on the same day (26 different individuals), we further considered the influence of proportion of time spent incubating on DEE (38 observation days) and water balance (39 observation days), fitting separate linear regressions for hot (≥35.5°C) and cool (<35.5°C) days. Individual identity was included as a random factor for all DEE and water balance analyses. The inclusion of nest or group identity as a random term in addition to individual identity resulted in unstable models and, of the two random terms, individual identity explained the greatest proportion of variation while avoiding destabilizing the models (Grueber *et al.*, 2011; Harrison *et al.*, 2018).

To determine which variables predicted ∆M_b_, we used the package *segmented* (Muggeo, 2008) to identify the temperature threshold (‘breakpoint’) above which ability to maintain body mass between days was compromised, followed by separate LMMs for the data above and below the breakpoint. For each model segment, we considered the influence of breeding season, T_max_, Sol_max_, Wind_max_, AbsHum_Tmax_, group size, sex, rank and the interactions between breeding season and group size and T_max_ and group size, with nest identity included as a random factor.

## Results

### Nest outcomes

**Figure 1:**
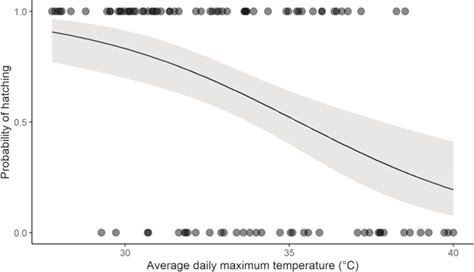
Nest outcomes as a function of mean daily maximum temperatures during incubation with the line showing the model fit and grey shaded area the 95% confidence interval (data from 99 nests by 23 southern pied babbler *T. bicolor* groups over 3 breeding seasons).

Of 99 nests monitored over 3 breeding seasons, 61 hatched and 38 failed. Mean T_maxInc_ was the most parsimonious predictor of variation in hatching success in pied babblers (the single best-fit model had a model weight of 0.794), and pied babbler nests were less likely to hatch as Mean T_maxInc_ experienced during incubation increased (Est = −0.949 ± 0.254, 95% CI: −1.479 to −0.477, *z* = −3.744, conditional R^2^ = 0.215; Fig. 1; see Supporting Information Table S4 for full model outputs). When Mean T_maxInc_ exceeded 35.3°C during incubation, the probability of pied babbler nests hatching dropped below 50%.

**Figure 2:**
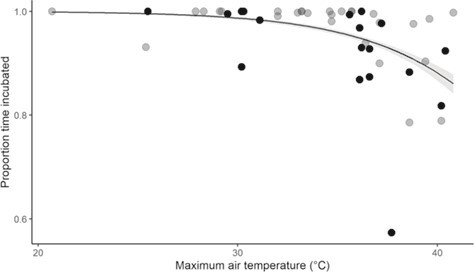
Proportion of time that the clutch was incubated as a function of maximum air temperature on the observation day with the line showing the model fit and grey shaded area the 95% confidence Interval, grey circles showing data collected one during the incubation period and black circles showing data collected on multiple days with a single incubation period. (data from 46 observation days at 35 southern pied babbler *T. bicolor* nests by 15 groups over 3 breeding seasons).

### Nest attendance

The percentage of time between dawn and 19 h00 that clutches were incubated ranged from 57.3 to 100% (median = 99%). Only 3 nests were incubated for <80% of daylight hours, all of which were observed on days with T_max_ > 37°C and all of which ultimately failed. T_max_ was the most parsimonious predictor of variation in the proportion of time that clutches were incubated. The single best-fit model (T_max_) had a model weight of 0.898 and the percentage of time clutches were incubated declined as temperatures increased (Est = −1.650 ± 0.492, 95% CI: −2.780 to −0.754, *z* = −3.355, conditional R^2^ = 0.998; Fig. 2; see Supporting Information Table S5 for full model ouputs). Additionally, the number of times clutches were left unattended per day (Fig. S2; Table S6), the proportion of time clutches were left unattended per day (Fig. S3; Table S7) and the probability of observing clutches that were left unattended at all (Fig. S4; Table S8) all increased as T_max_ increased.

### Nest temperatures

**Figure 3:**
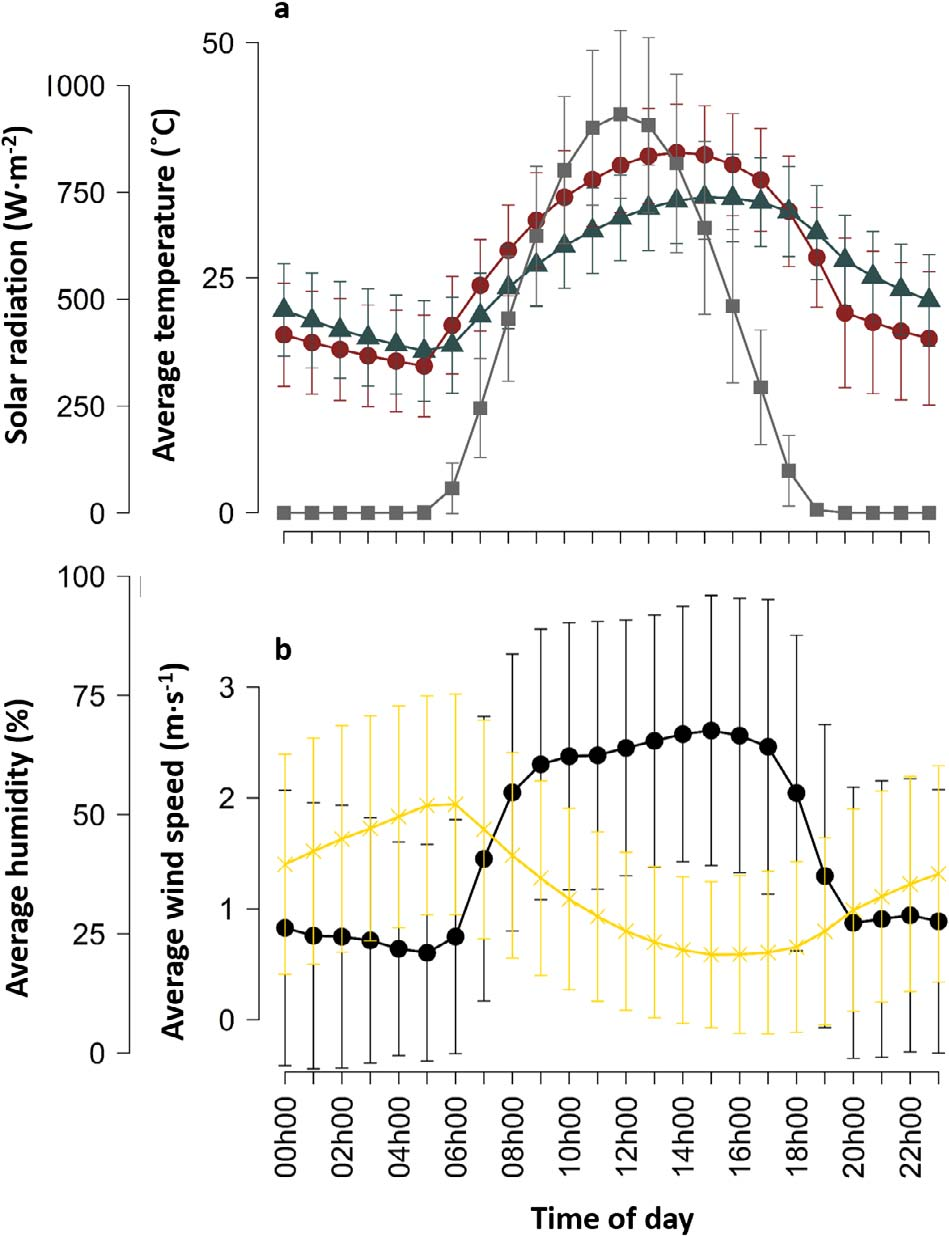
Comparison of (a) black bulb thermometers placed in vacated southern pied babbler *T. bicolor* nests (red circles) and average temperatures station (blue triangles) and solar radiation (grey squares) recorded per hour (mean±sd) by an onsite weather station, and (b) wind speed (black circles) and relative humidity (yellow crosses) recorded by the on-site weather station.

Diurnal nest T_e_ always exceeded T_air_ (06 h00–19 h00; mean difference = 7.9 ± 11.2°C; range, 0.0–31.8°C; Fig. 3a). At the coolest T_air_ recorded during the day (~8°C, *n* = 2 days), nest T_e_ averaged 10.1 ± 0.7°C (range, 8.8–11.6°C; *n* = 5 nests), and at the warmest T_air_ recorded during the day (~41°C, *n* = 1 day), nest T_e_ averaged 44.4 ± 2.8°C (range, 40.9–49.1°C; *n* = 1 nest). Individual nests could be up to 25°C hotter than other nests for the same T_air_ of ~ 35.5°C, identified as a critical temperature threshold for body mass maintenance, hatching success and parental care behaviour in pied babblers (Bourne *et al.*, 2020a; du Plessis *et al.*, 2012; Wiley and Ridley, 2016).

**Figure 4:**
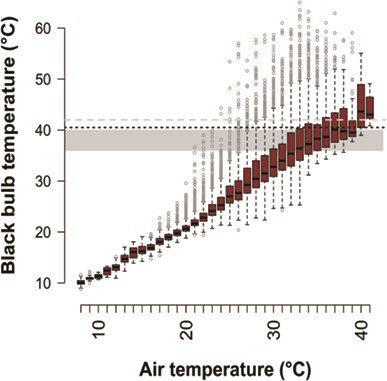
Black bulb thermometer temperature as a function of air temperature with boxplots showing the median and interquartile range (IQR) of operative temperature for each air temperature value rounded to the nearest digit; whiskers indicate the lowest and highest value data points within 1.5*IQR; points plotted beyond the whiskers represent a relatively small number of extreme values in this large dataset of 21 872 temperature records; the optimal temperature range for avian embryo development (36°C–40°C, shaded area), the lowest potentially lethal temperature for avian embryos given prolonged exposure (40.5°C, black dotted line) and the average upper critical limit for thermoneutrality in passerines (41°C, grey dashed line) are indicated.

Nest T_e_ increased significantly with T_air_ (linear regression; Est = 1.207 ± 0.005, 95% CI: 1.196 to 1.217, t = 229.2, Adj R^2^ = 0.83; Fig. 4). The highest nest T_e_ recorded was 65°C and operative temperatures >60°C were recorded at 2 nests for T_air_ between ~30°C and ~37°C. We recorded 2379 instances of T_e_ in unattended nests >41°C (10.8% of all T_e_ records, 22 of 23 nests; mean = 108 ± 85 instances per nest; range, 30–295), identified as a potentially lethal temperature for avian embryos (DuRant *et al.*, 2013; Webb, 1987). We further recorded 487 instances of T_e_ in unattended nests >50°C (2.2% of all T_e_ records, 14 of 23 nests; median = 3 instances per nest; range, 1–163), known to be lethal for the embryos of many arid-zone species (Grant, 1982; Griffith *et al.*, 2016; Reyna and Burggren, 2012).

### Energy expenditure and water balance

**Figure 5:**
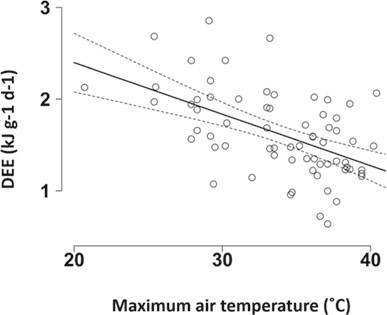
Variation in DEE by maximum air temperature (°C) on the measurement day in southern pied babblers T. bicolor, with the line showing the model fit and the dotted lines are the 95% confidence interval (data from 68 measurements of 45 different birds from 17 groups incubating 34 different clutches).

**Figure 6:**
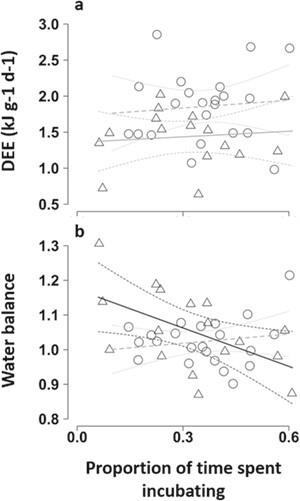
Relationship between proportion of time southern pied babblers *T. bicolor* spent incubating on cool (T_max_ < 35.5°C, open circles, dashed lines, dotted 95% CIs) and hot (T_max_ ≥ 35.5°C, open triangles, solid lines, dashed 95% CIs) days on the (a) daily energy expenditure and (b) water balance of incubating birds with the line showing the model fit and dashed lines representing 95% confidence interval; model fit lines for non-significant relationships are faded to grey.

We quantified DEE (*n* = 68; mean = 1.6 ± 0.5 kJ^−1^g^−1^d; range, 0.6 to 2.9 kJ^−1^g^−1^d) and water balance (*n* = 69; mean = 1.0 ± 0.1; range, 0.9 to 1.7; where 1 = neutral water balance) in 45 different birds from 17 groups incubating 34 different clutches. T_max_ was the most parsimonious predictor of variation in DEE (of two competing top models, the best-fit model had T_max_ as the only predictor and a model weight of 0.549), and DEE declined with increasing temperature (Est = −0.222 ± 0.046, 95% CI: −0.315 to −0.129, *z* = 4.694, conditional R^2^ = 0.557; Fig. 5; see Supporting Information Table S9 for full model ouput). Variation in water balance was not predicted by any of the variables included in our models (Table S10). Our within-individual physiology and behaviour data showed no evidence that DEE was predicted by the proportion of time spent incubating on either hot or cool days (*n* = 38; Fig. 6a; Table 1). However, these data showed that pied babblers’ ability to maintain neutral or positive water balance declined with an increasing proportion of time spent incubating on hot days, but not on cool days (*n* = 39; Fig. 6b; Table 1). Average metabolic water production declined from 4.7 g d^−1^ for T_max_ < 26°C to 2.8 g d^−1^ for T_max_ > 39°C. To make up that deficit from pre-formed water in order to maintain water balance, which they failed to do under the highest temperatures, pied babblers would have had to eat the equivalent of an extra 17 beetle larvae during the course of the day.

**Table 1:** DEE and water balance as a function of proportion of time spent incubating, overall and analysed separately for cool (T_max_ < 35.5°C) and hot (T_max_ ≥ 35.5°C) days

Response	*n*	Temperature	Estimate	Std error	95% CI	*t* value	*P*-value
DEE	38	Overall	0.564	0.578	−0.607/1.736	0.977	0.335
	22	Cool	0.360	0.871	−1.456/2.177	0.414	0.684
	16	Hot	0.258	0.662	−1.162/1.678	0.390	0.703
Water balance	39	Overall	−0.188	0.099	−0.388/0.012	−1.901	0.065
	22	Cool	0.089	0.117	−0.155/0.332	0.758	0.457
	**17**	**Hot**	**−0.369**	**0.149**	**−0.687/−0.052**	**−2.480**	**0.026**

Significant relationships are shown in boldface.

### Body mass

**Figure 7:**
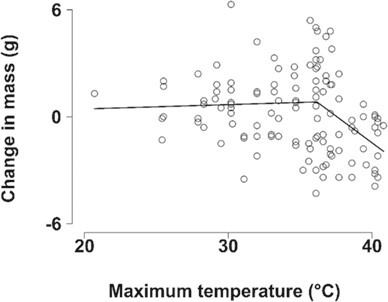
Change in southern pied babbler *T. bicolor* body mass (g) from one morning to the next as a function of maximum air temperature (°C) on the observation day with the line showing the segmented linear regressions for the relationship between mass change and temperature above and below the detected temperature threshold (36.1°C), i.e. no relationship below the threshold temperature and a significant negative relationship above the temperature threshold.

Mass change over 24 hours averaged 0.3 ± 2.2 g (range, −4.3 to 6.3 g; *n* = 119 individuals). Depending on starting body mass and mass change in grams, mass change ranged from −5.8 to 8.5% of body mass. We detected a threshold T_max_ at 36.1°C (95% CI: 33.9 to 38.3°C). At T_max_ < 36.1°C (*n* = 59), ∆M_b_ was not influenced by any of the predictor terms (Table S11). At T_max_ ≥ 36.1°C (*n* = 60), T_max_ was the only predictor that significantly influenced ∆M_b_ (model weight = 0.647), with mass loss becoming more likely as temperatures increased (Est = −1.016 ± 0.301, 95% CI: −1.605 to −0.427, *t* = −3.379, conditional R^2^ = 0.162; Fig. 7; see Supporting Information Table S12 for full model outputs).

## Discussion

Pied babblers exhibit poor hatching success at high temperatures (Bourne *et al.*, 2020a). Employing a novel combination of non-invasive DLW, nest temperature data and field-based behaviour observations, we demonstrated that pied babblers generally incubated their nests almost constantly (99% of daylight hours), but the proportion of time that nests were attended declined with increasing T_air_ (as has also been observed in blue *Cyanistes caeruleus* and great tits *Parus major*Bueno-Enciso *et al.*, 2017 and king rails (*Rallus elegans*) Clauser and McRae, 2017). Second, we found that operative temperatures in unattended nests frequently exceeded widely reported lethal limits for avian embryos (Birkhead *et al.*, 2008; Conway and Martin, 2000; DuRant *et al.*, 2013; Wada *et al.*, 2015; Webb, 1987) and the inflection air temperature values above which passerine birds rapidly increase rates of evaporative water loss via panting (McKechnie *et al.*, 2017; Smith *et al.*, 2017). Third, we found that pied babblers incurred water costs associated with incubation at high temperatures but energy expenditure did not increase with an increase in proportion of time spent incubating at high temperature (similar to recent studies of zebra finches *Taeniopygia guttata*Cooper *et al.*, 2019 and white-browed sparrow-weavers *Plocepasser mahali*Smit and McKechnie, 2015). Finally, we found that pied babblers from incubating groups lost mass during very hot weather [known to occur in pied babblers (du Plessis *et al.*, 2012) and other arid-zone bird species (Sharpe *et al.*, 2019; van de Ven *et al.*, 2019)]. In this study, mass loss occurred at T_max_ > 36.2°C, which is very similar to the threshold temperature for mass loss in pied babblers of 35.5°C previously identified in subordinate individuals in non-breeding groups at the same study site (du Plessis *et al.*, 2012). With T_e_ in unattended nests regularly exceeding lethal limits for avian embryos, reduced nest attendance at high T_air_ may contribute to reduced hatching success during hot incubation periods.

Our finding that pied babblers showed significant declines in DEE at high T_air_ is consistent with results from other studies (Cooper *et al.* 2019; Smit and McKechnie 2015) and likely reflects a decrease in activity as birds rest or seek shade at high air temperatures (Pattinson *et al.*, 2020; Pattinson and Smit, 2017; van de Ven *et al.*, 2019). An inflection point in metabolic rate would only be expected at environmental temperatures above the thermoneutral zone (McKechnie *et al.*, 2016, 2017; McKechnie and Wolf, 2019). However, our finding that incubating pied babblers failed, on average, to maintain water balance when incubating for long periods of time on hot days, but not on cool days, is novel and strongly suggests that birds incubating at high temperatures might leave the nest because of the water costs incurred from incubating in the heat (Bourne, 2020). In hot and dry environments such as the Kalahari Desert, incubating birds cannot fully engage in normal behavioural thermoregulation, such as retreating to the shade or adjusting foraging and drinking behaviours (Abdu *et al.*, 2018; Cooper *et al.*, 2019; Smit *et al.*, 2016). Incubating pied babblers do not eat while on the nest, instead alternating foraging bouts with incubation as all adult group members contributing to incubation throughout the day (Ridley and Raihani, 2007; Ridley and van den Heuvel, 2012), and thus are unlikely to gain additional water from food while on the nest. Using evaporative cooling to maintain body temperature below lethal levels (Brown and Downs, 2003; Grant, 1982; O’Connor *et al.*, 2018) presumably comes at high water cost to themselves given the high nest T_e_ we observed in pied babblers. Metabolic water production is generally too low to maintain water balance in hot environments (e.g. MacMillen 1990) and may be the reason that lethal dehydration has resulted in mass mortality of birds (Gardner *et al.*, 2019; McKechnie and Wolf, 2010) and mammals (Ratnayake *et al.*, 2019; Welbergen *et al.*, 2008) during heatwaves. The water turnover rates of birds in arid environments tend to be frugal (Cooper *et al.*, 2019; Williams and Tieleman, 2005). Those individuals that did maintain water balance when incubating for long periods at high temperatures may have been more successful while foraging during off bouts or had more food in their crops at the start of incubation (Conradie *et al.*, 2020).

The T_e_ we recorded in unattended pied babbler nests provided an index of the thermal environment likely experienced by incubating pied babblers in their nests and regularly exceeded (i) temperatures at which evaporative water loss increases rapidly in passerine birds (41°C; McKechnie *et al.*, 2017; Smith *et al.*, 2017), (ii) optimal temperatures for embryo development in passerines (36–40°C; DuRant *et al.*, 2013) and (iii) lethal temperature limits for developing avian embryos (40.5°C–51°C; DuRant *et al.*, 2013; Grant, 1982; Griffith *et al.*, 2016; Stoleson and Beissinger, 1999; Webb, 1987). Such high nest temperatures have been recorded in several bird species nesting in exposed sites and some arid-zone species exhibit quite high heat tolerance in developing embryos. For example, northern bobwhite *Colinus virginianus* eggs can survive exposure to temperatures of 46°C for about an hour (Reyna and Burggren, 2012). Nonetheless, leaving nests unattended for long periods of time during the heat of the day risks exposing developing avian embryos to high temperatures (Carroll *et al.*, 2015a; DuRant *et al.*, 2019; Mayer *et al.*, 2009), potentially exceeding lethal limits (Webb, 1987) and risking embryo death (Birkhead *et al.*, 2008; Clauser and McRae, 2017; Wada *et al.*, 2015) or leading to other problems such as an increased risk of nest predation (DeGregorio *et al.*, 2015). It is therefore likely that near-constant incubation and/or shading is both highly desirable (Grant, 1982), in order to limit exposure of embryos to excessive heat, and also difficult to sustain at high temperatures, because birds prevent body temperature exceeding lethal limits by evaporative cooling (Albright *et al.*, 2017; McKechnie and Wolf, 2019; O’Connor *et al.*, 2017). The reduced nest attendance we observed at high temperatures is consistent with a constraint on parental investment in incubation associated with the water costs of heat exposure (Amat and Masero, 2004; Coe *et al.*, 2015) and may suggest progress towards eventual nest abandonment (Bourne, 2020; Sharpe *et al.*, 2019; Stoleson and Beissinger, 1999). The T_e_ data we collected in nests clearly show that the T_air_ measurements used in analyses underestimated both the degree and the variability of heat exposure for incubating pied babblers on their nests. While we used T_air_ for analyses to increase comparability with other studies and improve the potential for our study to contribute towards climate impacts models, our findings suggest that future studies could benefit from recording T_e_ alongside T_air_. We were unable to test for a relationship between water balance and hatching success directly. We have anecdotal evidence of extended incubation recesses and signs of apparent dehydration in several birds after they had incubated for long periods of time on hot afternoons. In addition, we found at least one clutch that was definitely abandoned during the incubation phase following 5 consecutive days at >35.5°C (Bourne, 2020, reproduced in the supplementary materials). Reduced nest attendance on hot afternoons may suggest progress towards eventual nest abandonment (Clauser and McRae, 2017; Sharpe *et al.*, 2019; Stoleson and Beissinger, 1999). However, in most cases we were (i) unable to see the incubating bird clearly to enough to record detailed data on panting or shading behaviour (nests are often >5 m high); (ii) not able to consistently record behaviour data from the incubating bird because we had to follow and record behaviour observations from birds that were dosed with DLW in order to collect their faeces for the DLW analyses; (iii) not able to visit nests repeatedly during the incubation period due to other data collection commitments (breeding was often synchronous with other groups and we prioritized data from different individuals and nests over detailed data from within a smaller number of breeding attempts) and limitations of the DLW technique (e.g. the same individuals cannot be dosed again within ~2 weeks); and (iv) we were not always able to identify the precise cause of nest failure because, in most cases, we could not be sure if the nest had been abandoned or predated. Observed mass loss may well be associated with evaporative water loss, but to provide a comprehensive explanation of the underlying processes is beyond the scope of the current study. Future research could usefully explore the relationships among temperature, incubation effort, thermoregulatory behaviour and hydration status in birds in more detail.

### Conclusions

Given that (i) pied babblers incubate their eggs almost constantly during the day, (ii) lower incubation rates occurred on hot days and (iii) unusually low incubation constancy was often followed by nest abandonment or failure, we suggest that reduced incubation at high temperatures might contribute to hatching failure by increasing the risk of embryo exposure to lethal temperatures. We cannot directly test for causal relationships between effects of temperature on the behaviour and physiology of incubating pied babblers and hatching success, which would require an experimental approach or at least observations over multiple days within the same breeding attempts. However, we present multiple lines of evidence suggesting that pied babbler nests are more likely to hatch when incubated consistently. Ambient incubation at high operative temperatures may be detrimental to developing embryos, potentially exposing them to a greater risk of overheating (Cones, 2017). Incubating adults may be constrained from consistent incubation at high temperatures once thermoregulatory thresholds are approached or exceeded. We suggest that pied babblers may leave their nests on hot afternoons because incubating for prolonged periods at high temperatures may increase water costs. Considering both behaviour and physiology simultaneously in the same individuals, at the same time, under natural conditions, provides invaluable insights into the thermal constraints under which incubating birds operate. As we found no relationship between group size and any of the responses we measured, either alone or in interaction with environmental factors, we further suggest that cooperative breeding may not confer an advantage over non-cooperative breeding strategies in buffering against hot weather during the incubation phase. Future studies may usefully consider variation in the number of individuals that are actively involved in incubation rather than total group size.

Although parental care strategies are flexible in response to both climate and social conditions (Clutton-Brock *et al.*, 2004; Langmore *et al.*, 2016), these strategies have limits (Bourne *et al.*, 2021; Clauser and McRae, 2017; Sharpe *et al.*, 2019; van de Ven *et al.*, 2020). Given that both mean temperatures and hot extremes are increasing in frequency under global climate change (IPCC, 2013), the incubation period could become a major bottleneck for reproduction across species with different reproductive strategies. Birds will likely incur ever greater thermoregulatory costs of incubation as temperatures rise, leading to reduced nest attendance, potential overheating of eggs, and ultimately, compromised population replacement and persistence.

## Funding

This work was supported by the Australian Research Council (FT110100188 to A.R.R.), the BBSRC David Phillips Fellowship (BB/J014109/1 to C.N.S.), the British Ornithologists’ Union, the DST-NRF Centre of Excellence at the FitzPatrick Institute for African Ornithology, the Oppenheimer Memorial Trust (20747/01 to A.R.B.), the University of Cape Town and the National Research Foundation of South Africa (grant no. 110506 to A.E.M. and grant nos. 99050 and 118627 to S.J.C.).

## Author contributions

All authors conceived the study and secured funding. A.R.R. started habituation of the study animals in 2003 and has maintained it ever since, this was central to making the study possible; A.R.B. undertook all fieldwork with paid assistants; A.R.B. analysed the data and drafted the manuscript; all authors contributed substantially to revisions and gave final approval for publication.

## Data availability statement

The data underlying all analyses presented in this study have been archived at the University of Cape Town’s open access institutional data repository, ZivaHub (a figshare platform), where they are publicly available at doi:10.25375/uct.14499939.

## Supplementary Material

AppliedDLWms_SuppInfo_16052021_coab043
