# Supplementary material for: Dehydration risk is associated with reduced nest attendance and hatching success in a cooperatively breeding bird, the southern pied babbler Turdoides bicolor
Source: Conserv Physiol. 2021 Jun 16;9(1):coab043. doi: 10.1093/conphys/coab043 (PMC8208672; doi:10.1093/conphys/coab043)
Supplement: AppliedDLWms_SuppInfo_16052021_coab043 [file applieddlwms_suppinfo_16052021_coab043.docx]

# **Title: Dehydration risk is associated with reduced nest attendance and hatching success in a cooperatively breeding bird, the southern pied babbler *Turdoides bicolor***

# **Running head: Dehydration limits nest attendance**

Authors: Amanda R. Bourne*^1^, Amanda R. Ridley^1,2^, Andrew E. McKechnie^3,4^, Claire N. Spottiswoode^1,5^, Susan J. Cunningham^1^

*Corresponding author: Amanda Bourne [abourne.uct@gmail.com](mailto:abourne.uct@gmail.com)

^1^ FitzPatrick Institute of African Ornithology, DST-NRF Centre of Excellence, University of Cape Town, Private Bag X3, Rondebosch 7701, South Africa

^2^ Centre for Evolutionary Biology, School of Biological Sciences, University of Western Australia, Crawley 6009, Australia

^3^ South African Research Chair in Conservation Physiology, South African National Biodiversity Institute, Pretoria, South Africa

^4^ DST-NRF Centre of Excellence at the FitzPatrick Institute, Department of Zoology and Entomology, University of Pretoria, Hatfield, South Africa

^5^Department of Zoology, University of Cambridge, Downing Street, Cambridge CB2 3EJ, UK

**Supporting Information**

# **Weather conditions**

Weather conditions were not significantly different between the active nest period and the time periods during which T_e_ was recorded in the nests (Table S1, Figure S1). It was therefore reasonable to compare operative temperatures collected in pied babbler nests after the nests had been vacated by the birds with daily maximum air temperatures experienced by incubating birds during the time that the nest was active.


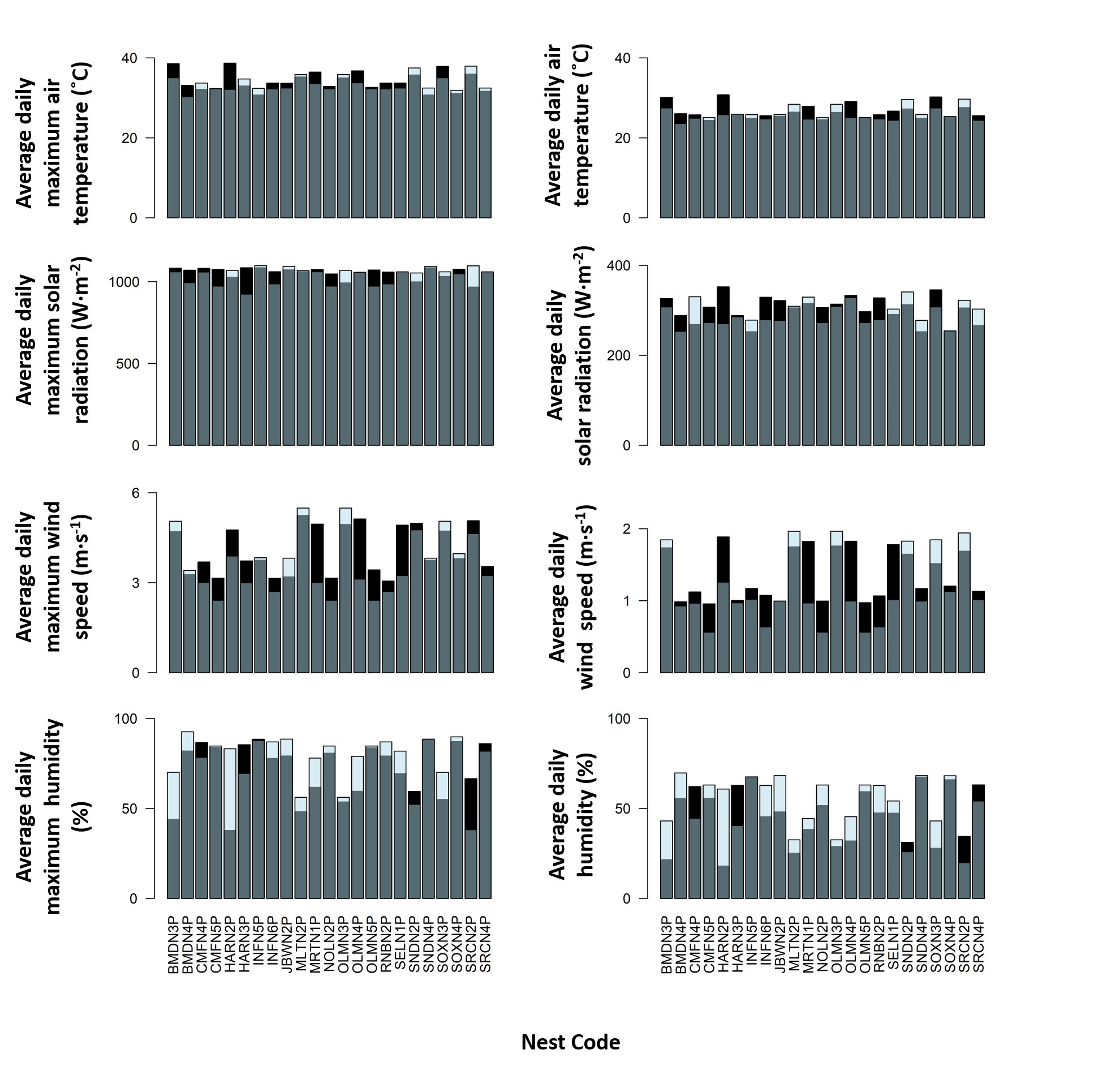


Figure S1: Average daily maximum air temperature (°C), average daily air temperature (°C), average daily maximum solar radiation (W·m^-2^), average daily solar radiation (W·m^-2^), average daily maximum wind speed (m·s^-1^), average daily wind speed (m·s^-1^), average daily maximum humidity (%) and average daily humidity (%) during the active nesting period (date on which incubation was initiated to date of hatching or nest failure, black bars) overlaid with the same measurements and during the time period in which nest operative temperatures were measured (date black bulb thermometer was installed to the date it was removed, light blue translucent bars – grey indicates overlap) for 23 nests.

Table S1: paired t-tests of average daily maximum and average daily weather during the active nesting period (date on which incubation was initiated to date of hatching or nest failure) and during the time period in which nest operative temperatures were measured (date black bulb thermometer was installed to the date it was removed) for 23 nests.

| **Weather variable** | ***df*** | ***t*** | ***p-value*** |
| --- | --- | --- | --- |
| Average daily maximum air temperature (°C) | 22 | 1.336 | 0.195 |
| Average daily air temperature (°C) | 22 | 1.391 | 0.178 |
| Average daily maximum solar radiation (W·m^-2^) | 22 | 1.101 | 0.283 |
| Average daily solar radiation (W·m^-2^) | 22 | 1.179 | 0.251 |
| Average daily maximum wind speed (m·s^-1^) | 22 | 1.679 | 0.137 |
| Average daily wind speed (m·s^-1^) | 22 | 1.725 | 0.124 |
| Average daily maximum humidity (%) | 22 | -1.807 | 0.084 |
| Average daily humidity (%) | 22 | -2.067 | 0.051 |

# **Mass**

**Table S2**

***Body mass measurements from 119 birds over three field seasons.***

| **Bird ID** | **Season Code** | **Mass 1** | **Mass 2** | **Mass Change** | **Change (proportion)** |
| --- | --- | --- | --- | --- | --- |
| XMYG | P | 78.8 | 78.3 | -0.5 | -0.01 |
| MYTT | P | 75.3 | 78.5 | 3.2 | 0.04 |
| MYTT | P | 70.4 | 68.3 | -2.1 | -0.03 |
| WMPY | P | 80.5 | 85.3 | 4.8 | 0.06 |
| HTML | P | 79.6 | 76.9 | -2.7 | -0.03 |
| CRMG | P | 80.4 | 79.8 | -0.6 | -0.01 |
| MSTY | P | 71.4 | 69.2 | -2.2 | -0.03 |
| MXLC | P | 73.4 | 77.2 | 3.8 | 0.05 |
| SOLM | P | 67.3 | 69.1 | 1.8 | 0.03 |
| GMGR | P | 82.9 | 81.9 | -1 | -0.01 |
| HTML | P | 76.5 | 77.9 | 1.4 | 0.02 |
| MSTY | P | 70.8 | 73.6 | 2.8 | 0.04 |
| MXLC | P | 75.9 | 75.8 | -0.1 | -0.00 |
| SOLM | P | 71.7 | 70.6 | -1.1 | -0.02 |
| GMSO | P | 77.2 | 80.1 | 2.9 | 0.04 |
| MRPP | P | 80.2 | 77.2 | -3 | -0.04 |
| BOOM | P | 80.5 | 81.4 | 0.9 | 0.01 |
| ROXM | P | 70.2 | 68.6 | -1.6 | -0.02 |
| SMBY | P | 77.8 | 76.4 | -1.4 | -0.02 |
| CHXM | P | 82.8 | 83.3 | 0.5 | 0.01 |
| PMPG | P | 79.1 | 79.9 | 0.8 | 0.01 |
| RLMP | P | 83.7 | 85.3 | 1.6 | 0.02 |
| MLTT | P | 77.5 | 78.9 | 1.4 | 0.02 |
| GMGR | P | 81.6 | 80.4 | -1.2 | -0.01 |
| HTML | P | 79.9 | 76.4 | -3.5 | -0.04 |
| XX18 | P | 78.8 | 77.7 | -1.1 | -0.01 |
| MYTT | Q | 73.5 | 71.3 | -2.2 | -0.03 |
| RMBS | Q | 73.9 | 73.4 | -0.5 | -0.07 |
| OMOO | Q | 81.3 | 80.5 | -0.8 | -0.01 |
| MLTT | Q | 74.2 | 70.8 | -3.4 | -0.05 |
| RRXM | Q | 77.7 | 75.8 | -1.9 | -0.02 |
| TOMX | Q | 75.2 | 76.2 | 1 | 0.01 |
| WMTR | Q | 65.9 | 68.3 | 2.4 | 0.04 |
| GLMY | Q | 63.9 | 62.9 | -1 | -0.02 |
| WMPY | Q | 79.2 | 80.6 | 1.4 | 0.02 |
| MXLR | Q | 81.8 | 80.7 | -1.1 | -0.01 |
| GOLM | Q | 76.4 | 77.7 | 1.3 | 0.02 |
| BOMC | Q | 69 | 73.2 | 4.2 | 0.06 |
| MXTY | Q | 76.9 | 74.2 | -2.7 | -0.04 |
| POML | Q | 74.6 | 76.6 | 2 | 0.03 |
| TMTT | Q | 74.6 | 79.1 | 4.5 | 0.06 |
| YYMO | Q | 70.8 | 73.1 | 2.3 | 0.03 |
| MBCT | Q | 69.1 | 66.3 | -2.8 | -0.04 |
| MRPP | Q | 75.1 | 71.7 | -3.4 | -0.05 |
| XXX6 | Q | 78.2 | 76.8 | -1.4 | -0.02 |
| MLTT | Q | 79.2 | 77.4 | -1.8 | -0.02 |
| TOMX | Q | 75.4 | 80.8 | 5.4 | 0.07 |
| BYMX | Q | 76.6 | 79.9 | 3.3 | 0.04 |
| MHWC | Q | 86.8 | 88.6 | 1.8 | 0.02 |
| CHYM | Q | 68.7 | 68.9 | 0.2 | 0.00 |
| MBCT | Q | 68.5 | 70.4 | 1.9 | 0.03 |
| MRPP | Q | 71.8 | 72.6 | 0.8 | 0.01 |
| XXX6 | Q | 73.8 | 80.1 | 6.3 | 0.09 |
| OHMY | Q | 80.2 | 81.2 | 1 | 0.01 |
| MXOR | Q | 82.2 | 80.9 | -1.3 | -0.02 |
| PMCL | Q | 74.9 | 74.8 | -0.1 | -0.00 |
| CRMG | Q | 82.9 | 84.2 | 1.3 | 0.02 |
| BLMB | R | 68.6 | 73.9 | 5.3 | 0.08 |
| STMG | R | 82.3 | 80.5 | -1.8 | -0.02 |
| XMYG | R | 78.2 | 78.9 | 0.7 | 0.01 |
| PRMP | R | 75.8 | 75.8 | 0 | 0.00 |
| BLMB | R | 76.8 | 73.4 | -3.4 | -0.04 |
| STMG | R | 81.2 | 81 | -0.2 | -0.00 |
| XMYG | R | 79.6 | 77 | -2.6 | -0.03 |
| BOMC | R | 73.5 | 74.6 | 1.1 | 0.01 |
| GMCY | R | 66.8 | 70.5 | 3.7 | 0.06 |
| BRXM | R | 76.5 | 78.3 | 1.8 | 0.02 |
| OMOO | R | 91.6 | 89.8 | -1.8 | -0.02 |
| PCMX | R |  |  | 0 |  |
| MXLR | R | 77.6 | 76.4 | -1.2 | -0.02 |
| PYMH | R | 74.1 | 69.8 | -4.3 | -0.06 |
| WMPY | R | 76.8 | 78.8 | 2 | 0.03 |
| CHMP | R | 68.6 | 69.2 | 0.6 | 0.01 |
| RMBS | R | 78.4 | 75.4 | -3 | -0.04 |
| STMG | R | 77.5 | 82.3 | 4.8 | 0.06 |
| PYMH | R | 71.1 | 71.1 | 0 | 0.00 |
| WMPY | R | 79.5 | 76.5 | -3 | -0.04 |
| BOMC | R | 73.3 | 72.7 | -0.6 | -0.01 |
| RMBS | R | 74.7 | 71.5 | -3.2 | -0.04 |
| CHMP | R | 70.5 | 70.6 | 0.1 | 0.00 |
| GMCY | R | 68.2 | 67.3 | -0.9 | -0.01 |
| PYMH | R | 69.3 | 69 | -0.3 | -0.00 |
| BOMC | R | 71.7 | 69.5 | -2.2 | -0.03 |
| RMBS | R | 72.2 | 72.1 | -0.1 | -0.00 |
| CHMP | R | 66.9 | 63.5 | -3.4 | -0.05 |
| BOMC | R | 71.1 | 74.3 | 3.2 | 0.05 |
| OHMY | R | 81.8 | 79.3 | -2.5 | -0.03 |
| TMYH | R | 76.8 | 75.3 | -1.5 | -0.02 |
| CHXM | R | 83.8 | 86.6 | 2.8 | 0.03 |
| RLMP | R | 80.9 | 83.2 | 2.3 | 0.03 |
| GLMY | R | 68.6 | 67.5 | -1.1 | -0.02 |
| MLTT | R | 77.3 | 77.2 | -0.1 | -0.00 |
| RRXM | R | 78.6 | 81.3 | 2.7 | 0.03 |
| WMTR | R | 72 | 72.9 | 0.9 | 0.01 |
| BLMG | R | 71.4 | 71.6 | 0.2 | 0.00 |
| MXLC | R | 76.9 | 75.8 | -1.1 | -0.01 |
| SOLM | R | 70.3 | 69.3 | -1 | -0.01 |
| GMCY | R | 66.8 | 71.8 | 5 | 0.07 |
| PYMH | R | 71.5 | 73 | 1.5 | 0.02 |
| WMPY | R | 75.4 | 79.1 | 3.7 | 0.05 |
| RMBS | R | 72 | 74.7 | 2.7 | 0.04 |
| CHMP | R | 65.4 | 69.1 | 3.7 | 0.06 |
| RRXM | R | 81.2 | 80.8 | -0.4 | -0.00 |
| BGMW | R | 75.8 | 76.3 | 0.5 | 0.01 |
| OHMY | R | 79.4 | 80.1 | 0.7 | 0.00 |
| TMYH | R | 74.8 | 76.3 | 1.5 | 0.02 |
| BGMW | R | 76.9 | 75.4 | -1.5 | -0.02 |
| OHMY | R | 82.1 | 82.6 | 0.5 | 0.01 |
| CLXM | R | 77.7 | 80.6 | 2.9 | 0.04 |
| LMCP | R | 86.2 | 88 | 1.8 | 0.02 |
| PHXM | R | 83.1 | 84.5 | 1.4 | 0.02 |
| BLMG | R | 75 | 74.4 | -0.6 | -0.01 |
| MXLC | R | 76.8 | 77.7 | 0.9 | 0.01 |
| SOLM | R | 71.5 | 72.2 | 0.7 | 0.01 |
| BGMW | R | 75.7 | 78.1 | 2.4 | 0.03 |
| OHMY | R | 81.2 | 81.1 | -0.1 | -0.00 |
| TMYH | R | 78.5 | 78.2 | -0.3 | -0.00 |
| GLMY | R | 71.1 | 71.1 | 0 | 0.00 |
| MLTT | R | 79.1 | 80.8 | 1.7 | 0.02 |
| WMTR | R | 73.3 | 75.3 | 2 | 0.03 |

# **Power to detect interactions**

Interactions between group size and climatic effects on development would be consistent with a buffering effect of group size on survival. We therefore conducted sensitivity power analyses to identify the minimum determinable effect of two-way interactions given our sample sizes (Cohen, 1988; Greenland et al., 2016), using the package *pwr* (Champely et al., 2018). For our regression models, we used the function **pwr.f2.test(u =,v =,f2 =,sig.level =,power =)**, where u = numerator degrees of freedom, v = denominator degrees of freedom, α (the signficance level; probability of finding an effect that is not there) = 0.05, and power (probability of finding an effect that is there) = 0.8. The value *f^2^* is the calculated value, representing the measure of determinable effect size. We assumed a fourfold increase in required sample size to adequately detect interactions in mixed-effects models (Leon & Heo, 2009), and found that two-way interaction effects would have to be moderate to very large for us to be able to detect them in this dataset (all Cohen’s *f^2^* > 0.21). We have sufficient sample size to detect a range of main effect sizes, from small to large, in all analyses (range *f^2^*: 0.04 – 0.18) – see Table S3 below. Cohen (1988) suggested that *f^2^* values of ~ 0.02, ~0.15, and ~0.35 represent small, medium, and large effect sizes respectively.

**Table S3**

***Power analyses for the interactions: multiple regression power calculations***

| **Analysis** | ***u*** | ***v*** | ***α*** | ***power*** | ***f^2^*** |
| --- | --- | --- | --- | --- | --- |
| **Nest outcomes** |  |  |  |  |  |
| Main effects | 2 | 99 | 0.05 | 0.8 | 0.081 |
| Interactions | 3 | 25 | 0.05 | 0.8 | 0.443 |
| **Nest attendance** |  |  |  |  |  |
| Main effects | 2 | 46 | 0.05 | 0.8 | 0.178 |
| Interactions | 3 | 12 | 0.05 | 0.8 | 1.142 |
| **Energy expenditure and water balance** |  |  |  |  |  |
| Main effects | 2 | 70 | 0.05 | 0.8 | 0.115 |
| Interactions | 3 | 17 | 0.05 | 0.8 | 0.658 |
| **Mass change** |  |  |  |  |  |
| Main effects | 2 | 120 | 0.05 | 0.8 | 0.066 |
| Interactions | 3 | 30 | 0.05 | 0.8 | 0.359 |

*u = model degrees of freedom; v = sample size, α = the significance level, and power (p) = probability of finding an effect that is there; f^2^ = measure of determinable effect size (values of ~ 0.02, ~0.15, and ~0.35 represent small, moderate, and large determinable effect sizes respectively).

# **Nest outcomes**

**Table S4**

| ***Effects of environmental and social factors on probability of hatching*** | | | |
| --- | --- | --- | --- |
|  |  |  |  |
| Data from 99 nests by 23 groups over 3 breeding seasons | | | |
| Random term: Nest identity  Data analysis: binomial glmer with binary response (hatch = 1, fail = 0) in *lme4* | | |  |
|  |  |  |  |
| *Model terms* | *AICc* | *∆AICc* | *weight* |
| Null model | 130.1 | 14.72 | 0.001 |
| Season | 133.6 | 18.24 | 0.000 |
| Mean T_maxInc_ | 115.4 | 0.00 | 0.794 |
| Mean Sol_maxInc_ | 132.1 | 16.76 | 0.000 |
| Mean Wind_maxInc_ | 132.1 | 16.77 | 0.000 |
| Mean AbsHum_TmaxInc_ | 132.1 | 16.68 | 0.000 |
| Group size | 131.7 | 16.33 | 0.000 |
| Group size + Group size^2 | 132.5 | 17.17 | 0.000 |
| Mean T_maxInc_ + Group size + Mean T_maxInc_ * Group size | 118.1 | 2.72 | 0.204 |
| Season + Group size + Season * Group size | 135.1 | 19.71 | 0.000 |
|  | *AICc* | *∆AICc* | *weight* |
| Null model | 129.9 | 16.80 | 0.00 |
| *Top models:* |  |  |  |
| Mean T_maxInc_ | 113.1 | 0.00 | 1.00 |
| Effect size of explanatory terms after model averaging | Estimate | SE | 95% CI |
| Intercept | 0.640 | 0.237 | 0.188/1.122 |
| **Mean T_maxInc_** | **-0.949** | **0.254** | **-1.4790/-0.477** |

# **Nest attendance**

**Table S5**

| ***Effects of environmental and social factors on proportion of time that clutches were incubated*** | | | |
| --- | --- | --- | --- |
|  |  |  |  |
| Data from 46 observation days at 35 nests by 15 groups over 3 breeding seasons | | | |
| Random term: Nest identity  Data analysis: binomial glmer with *cbind* function in *lme4* | | |  |
|  |  |  |  |
| *Model terms* | *AICc* | *∆AICc* | *weight* |
| Null model | 352.8 | 10.31 | 0.005 |
| Season | 356.5 | 14.53 | 0.000 |
| T_max_ | 342.4 | 0.00 | 0.898 |
| Sol_max_ | 355.1 | 12.67 | 0.002 |
| Wind_max_ | 353.6 | 11.17 | 0.003 |
| AbsHum_Tmax_ | 345.1 | 11.60 | 0.003 |
| Group size | 354.5 | 12.09 | 0.002 |
| Group size + Group size^2 | 350.6 | 8.14 | 0.015 |
| T_max_ + Group size + T_max_ * Group size | 347.5 | 5.08 | 0.071 |
| Season + Group size + Season * Group size | 362.6 | 20.19 | 0.000 |
|  | *AICc* | *∆AICc* | *weight* |
| Null model | 352.8 | 11.4 | 0.00 |
| *Top models:* |  |  |  |
| T_max_ | 341.42 | 0.00 | 1.00 |
| Effect size of explanatory terms after model averaging | Estimate | SE | 95% CI |
| Intercept | 5.748 | 0.559 | 4.761/7.088 |
| **T_max_** | **-1.650** | **0.492** | **-2.780/-0.754** |

Higher temperatures were also associated with more frequent incubation recesses (Est = 0.541 ± 0.233, 95% CI: 0.111, 1.056, *z* = 2.323; Fig. S2; Table S6), longer total durations of incubation recesses (Wilcoxon rank sum *W* = 890, *p* = 0.012 comparing durations between hot and cools days using 35.5˚C as the threshold; Fig. S3; Table S7), and a higher probability of observing any incubation recesses at all (Est = 1.498 ± 0.712, 95% CI: 0.061, 2.934, z = 2.043; Fig. S4; Table S8).

The babblers leave their nests unattended infrequently, averaging 2 ± 2 times a day where no group members are incubating the clutch (range: 0 – 9). After model averaging, only T_max_ significantly predicted the number of times that clutches were left unattended, with the number increasing as temperatures increased (Table S6, Figure S2). At T_max_ exceeding 35.5°C, identified as a critical temperature threshold in pied babblers (du Plessis et al. 2012; Wiley & Ridley 2016), the number of times that clutches were left unattended averaged 3 (± 2, range: 0 – 9), whereas on cool days (maximum temperates < 35.5°C) the number of times that clutches were left unattended averaged < 1(range: 0 – 8). We only recorded a number of times that clutches were left unattended exceeding 2 on cool days twice across three breeding seasons – both of these nests were the first nests of inexperienced pairs from groups primarily made up of siblings rather than offspring.

**Table S6**

| ***Effects of environmental and social factors on number of times a day that clutches were left completely unattended*** | | | |
| --- | --- | --- | --- |
|  |  |  |  |
| Data from 46 observation days at 35 nests by 15 groups over 3 breeding seasons | | | |
| Random term: Nest identity  Data analysis: poisson glmer in *lme4* | | |  |
|  |  |  |  |
| *Model terms* | *AICc* | *∆AICc* | *weight* |
| Null model | 162.3 | 3.74 | 0.070 |
| Season | 165.6 | 7.04 | 0.013 |
| T_max_ | 158.6 | 0.00 | 0.452 |
| Sol_max_ | 164.7 | 6.15 | 0.021 |
| Wind_max_ | 161.7 | 3.12 | 0.095 |
| AbsHum_Tmax_ | 164.6 | 6.02 | 0.022 |
| Group size | 164.3 | 5.73 | 0.026 |
| Group size + Group size^2 | 160.4 | 1.78 | 0.185 |
| T_max_ + AbsHum_max_ | 159.7 | 1.14 | 0.181 |
| T_max_ + Group size + T_max_ * Group size | 161.3 | 2.74 | 0.115 |
| Season + Group size + Season * Group size | 171.9 | 13.31 | 0.001 |
|  | *AICc* | *∆AICc* | *weight* |
| Null model | 162.30 | 3.70 | 0.00 |
| *Top models:* |  |  |  |
| T_max_ | 158.60 | 0.00 | 0.71 |
| Group size + Group size^2 | 160.38 | 1.78 | 0.29 |
| Effect size of explanatory terms after model averaging | Estimate | SE | 95% CI |
| Intercept | -0.251 | 0.449 | --1.145/0.642 |
| **T_max_** | **0.541** | **0.233** | **0.070/1.012** |
| Group size | -0.129 | 0.234 | -0.591/0.334 |
| Group size ^ 2 | 0.222 | 0.380 | -0.527/0.970 |


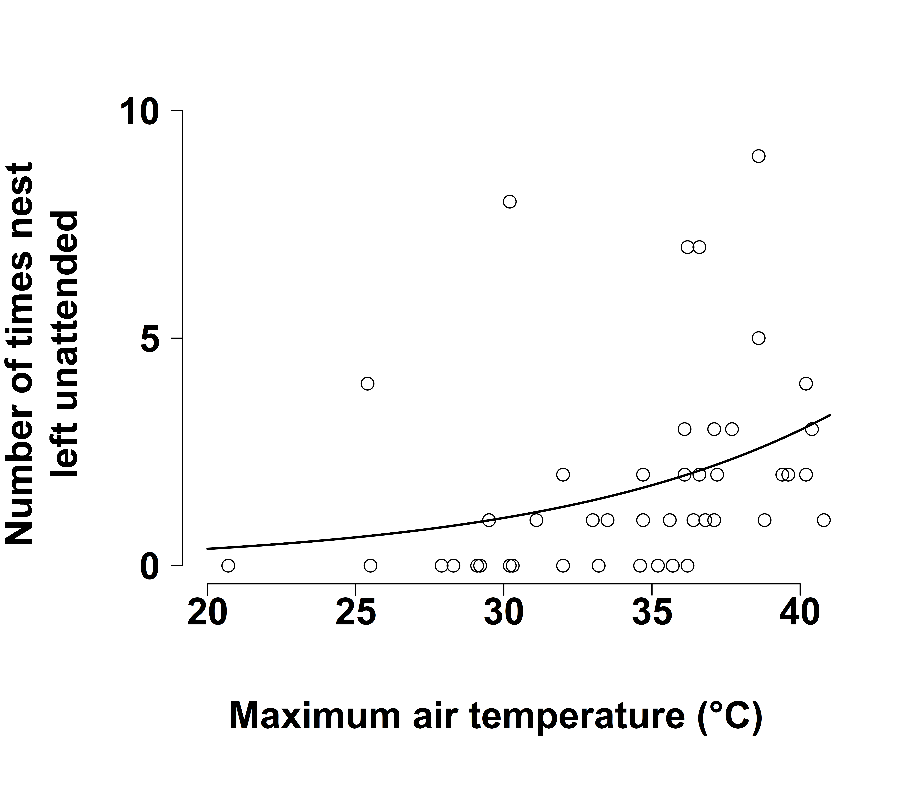


Figure S2: Number of times that the clutch was left unattended by maximum temperature on the observation day. Data from 46 observation days at 35 nests over 3 breeding seasons.

The duration of time periods during which clutches were left unattended was usually quite short (n = 83; median = 10 min, range: 1 – 265 min) – 80.7% of these periods were shorter than 30 min. The average duration of time periods during which clutches were left unattended was significantly longer on hot days (median = 13 min, range: 1 – 265 min) than on cool days (median = 5 min, range: 1 – 37 min; Wilcoxon rank sum *W* = 890, *p* = 0.012). Most of the time, clutches were left unattended for less than 30 min in total per day – we recorded clutch non-attendance totalling longer than 30 min per day on only 15 of 46 observation days. Thirteen of these occurred on days where maximum air temperatures exceeded 35.5°C and the other two were the nests of the inexperienced pairs from groups primarily made up of siblings rather than offspring described above. Days with no or very short total time periods for which clutches were left unattended (n = 24 with total non-attendance < 10 min) tended to be cooler (18 of 24 days had maximum temperatures < 35.5°C, mean = 33.1 ± 4.3°C; Wilcoxon rank sum *W* = 104, *p* = 0.003). These data are modelled as the inverse of nest attendance, with proportion of time that clutches were left unattended as the response and with the same results. The single top model, with model weight = 0.898, showed that only T_max_ significantly predicted the proportion of time that clutches were left unattended, with proportion time unattended increasing as temperatures increased (Table S7, Figure S3).

**Table S7**

| ***Effects of environmental and social factors on proportion of time that clutches were left completely unattended*** | | | |
| --- | --- | --- | --- |
|  |  |  |  |
| Data from 46 observation days at 35 nests by 15 groups over 3 breeding seasons | | | |
| Random term: Nest identity  Data analysis: binomial glmer with *cbind* function in *lme4* | | |  |
|  |  |  |  |
| *Model terms* | *AICc* | *∆AICc* | *weight* |
| Null model | 352.8 | 10.31 | 0.005 |
| Season | 356.5 | 14.09 | 0.001 |
| T_max_ | 342.4 | 0.00 | 0.898 |
| Sol_max_ | 355.1 | 12.67 | 0.002 |
| Wind_max_ | 353.6 | 11.17 | 0.003 |
| AbsHum_Tmax_ | 354.1 | 11.60 | 0.003 |
| Group size | 354.5 | 12.09 | 0.002 |
| Group size + Group size^2 | 350.6 | 8.14 | 0.015 |
| T_max_ + Group size + T_max_ * Group size | 347.5 | 5.08 | 0.071 |
| Season + Group size + Season * Group size | 362.6 | 20.19 | 0.000 |
|  | *AICc* | *∆AICc* | *weight* |
| Null model | 352.80 | 11.38 | 0.00 |
| *Top models:* |  |  |  |
| T_max_ | 341.42 | 0.44 | 1.00 |
| Effect size of explanatory terms after model averaging | Estimate | SE | 95% CI |
| Intercept | -5.748 | 0.559 | -7.088/-4.761 |
| **T_max_** | **1.650** | **0.492** | **0.754/2.779** |


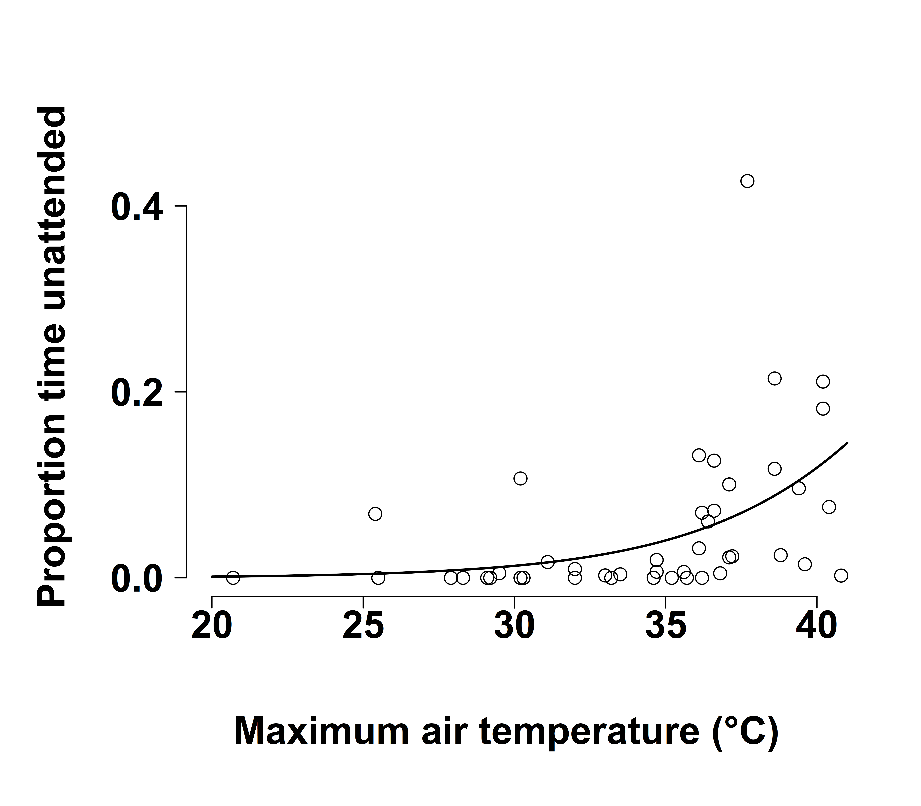


Figure S3: Proportion of time that the clutch was left unattended by maximum temperature on the observation day. Data from 46 observation days at 35 nests over 3 breeding seasons.

Clutches were not left unattended at all on 16 of the observation days. These days were all signficantly cooler (mean = 31.1 ± 4.3°C) than the days on which clutches were left unattended at least once (mean = 36.0 ± 3.6°C; Wilcoxon rank sum *W* = 75.5, *p* = 0.001, n = 31). The single top model (weight = 0.803) showed that only T_max_ significantly predicted the probability of observing that clutches were left unattended at all, with the probability of at least on period of non-attendance increasing as temperatures increased (Table S8, Figure S4). 78.6% of clutches that ultimately failed to hatch (n = 14) were left unattended at least once on our obervation days, whereas clutches that ultimately hatched (n = 21) were less likely to be left unattended - only 52.4% of were left unattended at least once on our observations days. The difference is not statistically significant (*X^2^_1_* = 1.473, *p* = 0.225).

**Table S8**

| ***Effects of environmental and social factors on the probability that clutches were left completely unattended at all*** | | | |
| --- | --- | --- | --- |
|  |  |  |  |
| Data from 46 observation days at 35 nests by 15 groups over 3 breeding seasons | | | |
| Random term: Nest identity  Data analysis: binomial glmer in *lme4* | | |  |
|  |  |  |  |
| *Model terms* | *AICc* | *∆AICc* | *weight* |
| Null model | 63.4 | 10.74 | 0.004 |
| Season | 66.2 | 13.57 | 0.001 |
| T_max_ | 52.7 | 0.00 | 0.803 |
| Sol_max_ | 64.3 | 11.64 | 0.002 |
| Wind_max_ | 65.5 | 12.82 | 0.001 |
| AbsHum_Tmax_ | 63.4 | 10.69 | 0.004 |
| Group size | 65.4 | 12.70 | 0.001 |
| Group size + Group size^2 | 59.7 | 6.98 | 0.024 |
| T_max_ + AbsHum_max_ | 52.7 | 0.00 | 0.438 |
| T_max_ + Group size + T_max_ * Group size | 55.9 | 3.24 | 0.159 |
| Season + Group size + Season * Group size | 73.7 | 21.07 | 0.000 |
|  | *AICc* | *∆AICc* | *weight* |
| Null model | 63.40 | 11.75 | 0.00 |
| *Top models:* |  |  |  |
| T_max_ | 51.65 | 0.00 | 1.00 |
| Effect size of explanatory terms after model averaging | Estimate | SE | 95% CI |
| Intercept | 0.742 | 0.492 | -0.254/1.860 |
| **T_max_** | **1.651** | **0.813** | **0.060/3.209** |


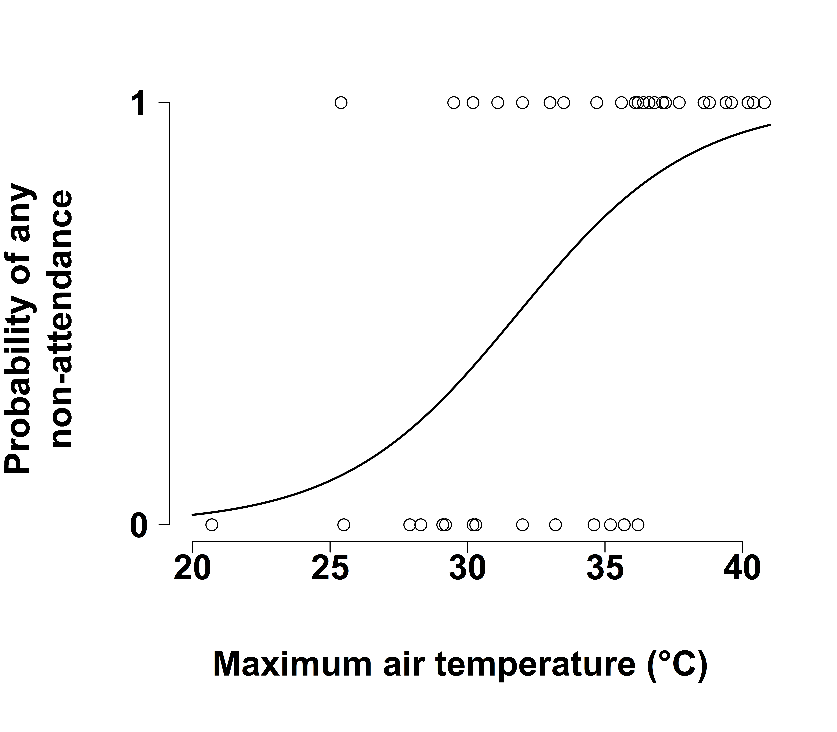


Figure S4: Whether or not a clutch was left unattended at all by maximum temperature on the observation day. Data from 46 observation days at 35 nests over 3 breeding seasons.

**Table S9**

| ***Effects of environmental and social factors on*** ***variation in daily energy expenditure in birds from groups incubating clutches*** | | | | | | | |
| --- | --- | --- | --- | --- | --- | --- | --- |
|  | |  | |  | |  | |
| Data from 68 observations on 45 different individuals at 34 nests by 17 groups over 3 breeding seasons | | | | | | | |
| Random term: Bird identity  Data analysis: Gaussian lmer in lme4 | | | | | |  | |
|  | |  | |  | |  | |
| *Model terms* | | *AICc* | | *∆AICc* | | *weight* | |
| Null model | | 91.6 | | 13.30 | | 0.001 | |
| Season | | 92.9 | | 14.62 | | 0.000 | |
| T_max_ | | 78.3 | | 0.00 | | 0.549 | |
| Sol_max_ | | 97.9 | | 19.64 | | 0.000 | |
| Wind_max_ | | 95.2 | | 16.97 | | 0.000 | |
| AbsHum_Tmax_ | | 93.6 | | 15.32 | | 0.000 | |
| Group size | | 90.9 | | 12.62 | | 0.001 | |
| Sex | | 94.3 | | 16.09 | | 0.000 | |
| Rank | | 95.5 | | 17.21 | | 0.000 | |
| T_max_ + Group size | | 82.4 | | 4.13 | | 0.070 | |
| T_max_ + Season | | 80.0 | | 1.70 | | 0.234 | |
| Group size + Season | | 89.2 | | 10.99 | | 0.002 | |
| T_max_ + Group size + Season | | 82.4 | | 4.12 | | 0.070 | |
| T_max_ + Group size + T_max_ * Group size | | 83.3 | | 5.03 | | 0.044 | |
| Season + Group size + Season * Group size | | 97.1 | | 1.83 | | 0.000 | |
| T_max_ + Group size + Season + T_max_ * Group size | | 87.0 | | 8.78 | | 0.007 | |
|  | | *AICc* | | *∆AICc* | | *weight* | |
| Null model | | 91.60 | | 13.34 | | 0.00 | |
| *Top models:* | |  | |  | |  | |
| T_max_ | | 78.26 | | 0.00 | | 0.70 | |
| T_max_ + Season | | 79.96 | | 1.70 | | 0.30 | |
| Effect size of explanatory terms | | Estimate | | SE | | 95% CI | |
| Intercept | | 1.551 | | 0.144 | | -1.270/1.834 | |
| **T_max_** | | **-0.222** | | **0.046** | | **-0.314/-0.129** | |
| Season (2016-17) | | 0.400 | | 0.135 | | -0.131/0.670 | |
| Season (2017-18) | | 0.120 | | 0.198 | | -0.287/0.508 | |
| Season (2018-19) | | 0.079 | | 0.139 | | -0.194/0.353 | |

**Table S10**

| ***Effects of environmental and social factors on variation in water balance in birds from groups incubating clutches*** | | | |
| --- | --- | --- | --- |
|  |  |  |  |
| Data from 69 observations on 45 different individuals at 34 nests by 17 groups over 3 breeding seasons | | | |
| Random term: Bird identity  Data analysis: Gaussian lmer in lme4 | | |  |
|  |  |  |  |
| *Model terms* | *AICc* | *∆AICc* | *weight* |
| Null model | -128.2 | 0.00 | 0.921 |
| Season | -114.8 | 13.47 | 0.001 |
| T_max_ | -118.7 | 9.57 | 0.008 |
| Sol_max_ | -118.7 | 9.55 | 0.008 |
| Wind_max_ | -119.0 | 9.26 | 0.009 |
| AbsHum_Tmax_ | -119.2 | 9.04 | 0.010 |
| Group size | -118.7 | 9.54 | 0.008 |
| Sex | -120.3 | 7.89 | 0.018 |
| Rank | -120.3 | 7.88 | 0.018 |
| T_max_ + Group size + T_max_ * Group size | -100.1 | 28.14 | 0.000 |
| Season + Group size + Season * Group size | -89.6 | 38.59 | 0.000 |
|  | *AICc* | *∆AICc* | *weight* |
| *Top models:* |  |  |  |
| Null model | -128.62 | 0.00 | 1.00 |
| Effect size of explanatory terms | Estimate | SE | 95% CI |
| Intercept | 1.027 | 0.010 | 1.007/1.048 |

**Table S11**

| ***Effects of environmental and social factors on*** ***mass change between days in individuals from groups incubating clutches (temperature < 36.1˚C)*** | | | |
| --- | --- | --- | --- |
|  |  |  |  |
| Data from 59 observations on 39 different individuals at 22 nests by 12 groups over 3 breeding seasons | | | |
| Random term: Nest identity  Data analysis: Gaussian lmer in *lme4* | | |  |
|  |  |  |  |
| *Model terms* | *AICc* | *∆AICc* | *weight* |
| Null model | 249.7 | 1.11 | 0.168 |
| Season | 248.6 | 0.00 | 0.293 |
| T_max_ | 252.5 | 3.91 | 0.042 |
| Sol_max_ | 252.3 | 3.73 | 0.045 |
| Wind_max_ | 250.1 | 1.58 | 0.133 |
| AbsHum_Tmax_ | 251.0 | 2.43 | 0.087 |
| Group size | 252.5 | 3.95 | 0.041 |
| Sex | 251.5 | 2.96 | 0.067 |
| Rank | 251.5 | 2.90 | 0.069 |
| T_max_ + Group size + T_max_ * Group size | 257.9 | 9.37 | 0.003 |
| Season + Group size + Season * Group size | 252.0 | 3.46 | 0.052 |
|  | *AICc* | *∆AICc* | *weight* |
| *Top models:* |  |  |  |
| Season | 248.56 | 0.00 | 0.49 |
| Null model | 249.67 | 1.11 | 0.28 |
| Wind Speed_max_ | 250.14 | 1.58 | 0.23 |
| Effect size of explanatory terms | Estimate | SE | 95% CI |
| Intercept | 0.307 | 0.367 | -0.417/1.031 |
| Season (2016-17) | -0.136 | 0.429 | -0.994/0.721 |
| Season (2017-18) | 0.613 | 0.710 | -0.787/2.012 |
| Season (2018-19) | 0.298 | 0.409 | -0.417/1.031 |
| Wind Speed_max_ | 0.102 | 0.232 | -0.357/0.561 |

**Table S12**

| ***Effects of environmental and social factors on mass change between days in individuals from groups incubating clutches (temperature ≥ 36.1˚C)*** | | | |
| --- | --- | --- | --- |
|  |  |  |  |
| Data from 60 observations of 32 different individuals at 14 nests by 10 groups over 3 breeding seasons | | | |
| Random term: Nest identity  Data analysis: Gaussian lmer in *lme4* | | |  |
|  |  |  |  |
| *Model terms* | *AICc* | *∆AICc* | *weight* |
| Null model | 286.3 | 7.73 | 0.014 |
| Season | 287.3 | 8.72 | 0.008 |
| T_max_ | 278.6 | 0.00 | 0.647 |
| Sol_max_ | 281.0 | 2.42 | 0.193 |
| Wind_max_ | 286.0 | 7.35 | 0.016 |
| AbsHum_Tmax_ | 287.1 | 8.53 | 0.009 |
| Group size | 288.7 | 10.07 | 0.004 |
| Sex | 287.3 | 8.73 | .008 |
| Rank | 287.6 | 9.00 | 0.007 |
| T_max_ + Group size + T_max_ * Group size | 282.5 | 3.90 | 0.092 |
| Season + Group size + Season * Group size | 290.9 | 12.28 | 0.001 |
|  | *AICc* | *∆AICc* | *weight* |
| Null model | 286.3 | 8.40 | 0.00 |
| *Top model:* |  |  |  |
| T_max_ | 277.9 | 0.00 | 1.00 |
| Effect size of explanatory terms | Estimate | SE | 95% CI |
| Intercept | 0.085 | 0.298 | -0.578/0.668 |
| **T_max_** | **-1.016** | **0.301** | **-1.605/-0.427** |

# **Anecdotal evidence of dehydration after prolonged incubation at high temperatures**

Reproduced with minor editorial adjustments from: Bourne, Amanda Ruth. 2020. Apparent dehydration in incubating southern pied babblers *Turdoides bicolor* Promerops 318: 12-14 available online at www.capebirdclub.org.za

Apparent dehydration in incubating southern pied babblers *Turdoides bicolor*

# Abstract

Incubation, which maintains egg temperatures within the optimum range for embryo development, could be costly for birds in hot and dry environments. We provide three examples of apparent (non-lethal) dehydration in southern pied babblers *Turdoides bicolor* observed incubating for long periods of time on hot days.

# Introduction

The incubation period may be a major bottleneck in avian reproduction. Hatching failure is widespread (Hemmings & Birkhead, 2015) and is particularly common at high temperatures (Clauser & McRae, 2017; Wada et al., 2015) and during droughts (Conrey, Skagen, Yackel Adams, & Panjabi, 2016). One of the reasons for hatching failure in hot and dry environments might be that birds incubating at high temperatures suffer thermoregulatory costs (Bueno-Enciso, Barrientos, Ferrer, & Sanz, 2017; Clauser & McRae, 2017) that force them to trade off incubation constancy against their own thermoregulation (DuRant, Willson, & Carroll, 2019; McKechnie, 2019; O’Connor, Brigham, & McKechnie, 2018). Here, we provide three examples of apparent (non-lethal) dehydration observed in southern pied babblers *Turdoides bicolor* (hereafter ‘pied babblers’) incubating at high temperatures.

# Methods and Materials

Examples of apparent dehydration in incubating pied babblers were collected in an ad hoc manner during full days of behavioural observation on which the authors waited near the nest at dawn, observed the first bird to replace the dominant female in the morning, and subsequently walked with the group all day recording incubation behaviour until 19h00. The study animals were habituated but free-living pied babblers (Ridley, 2016) at the 33 km2 Kuruman River Reserve (KRR; 26°58’S, 21°49’E) in the southern Kalahari. Pied babblers are medium-sized (60–90 g), cooperatively-breeding passerines that live in groups ranging in size from 3–15 adults and are endemic to the region (Ridley, 2016). Birds in the study population are individually identifiable by unique combinations of metal and colour rings fastened to their legs as nestlings (Fig.1), and are habituated to observation by humans from distances of 1-5 m (Ridley, 2016). All individuals referred to here were of known sex and rank (Nelson-Flower et al., 2011; Wiley & Ridley, 2018). Measurements of daily maximum air temperature were obtained from an onsite weather station (Vantage Pro2, Davis Instruments, Hayward, USA). Location of nests and monitoring of nest outcomes followed Ridley and van den Heuvel (2012). Pied babblers build open cup nests, usually in camelthorn *Vachellia erioloba* trees (Fig. 2), and tend to breed in summer when it is hottest (Ridley, 2016). Only the dominant female incubates overnight (Ridley, 2016), so we recorded incubation behaviour during daylight hours, when all group members take turns to incubate (Ridley & Raihani, 2007; Ridley & van den Heuvel, 2012). Generally speaking, clutches of eggs are rarely left unattended in this species (Ridley & Thompson, 2011). Body mass measurements were obtained at dawn by enticing birds to hop onto a top-pan balance in exchange for a small food reward (Ridley, 2016). Water balance measurements were obtained using a non-invasive doubly-labelled water technique (Bourne et al., 2019) and Nagy’s (1980) equations.


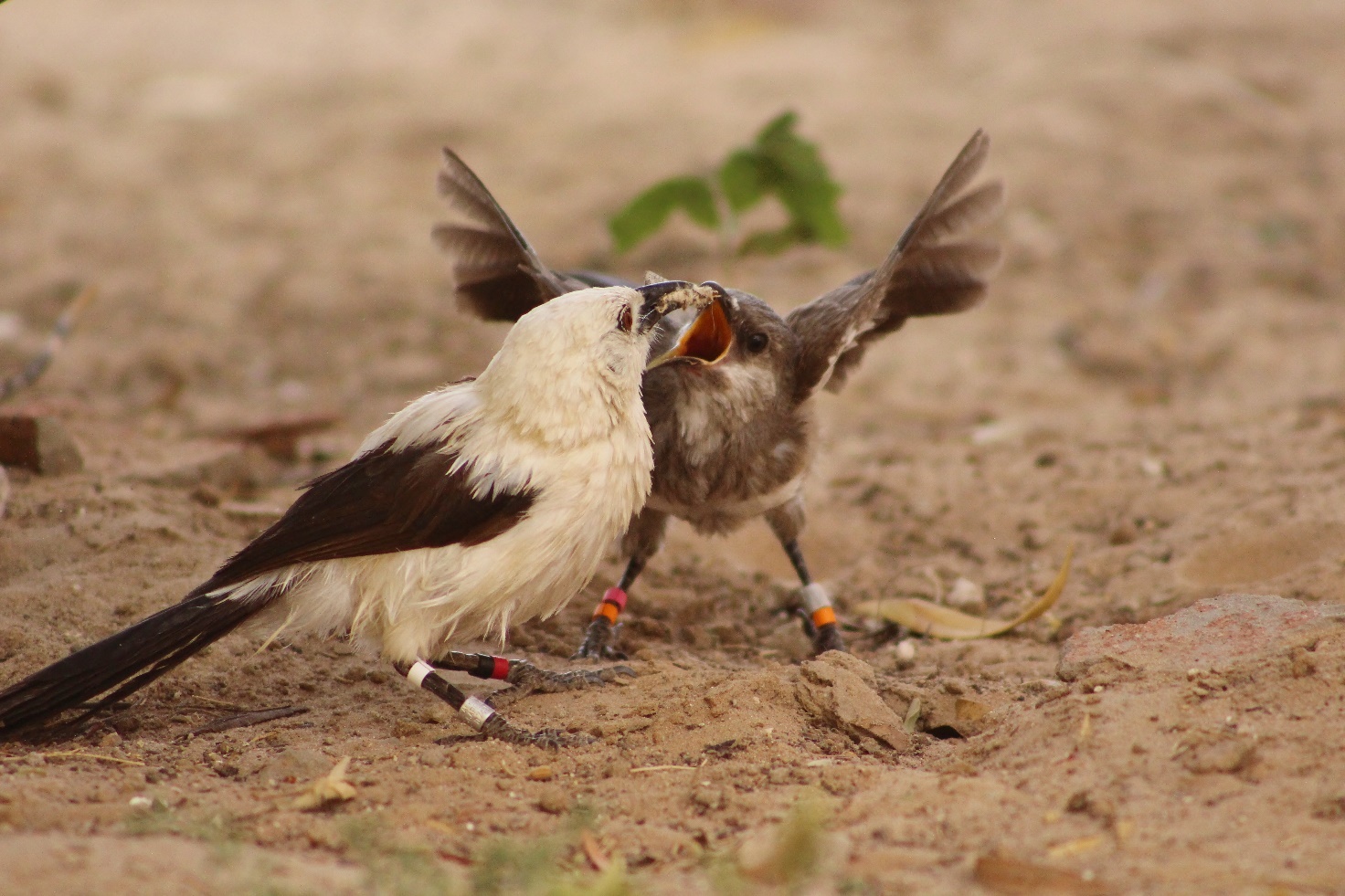


Figure 1: Southern Pied Babblers *Turdoides bicolor* are cooperative breeders. In this picture, a subordinate female feeds a young fledgling. Individuals in the study population are uniquely identifiable by their metal and colour ring combinations. © Nicholas B. Pattinson


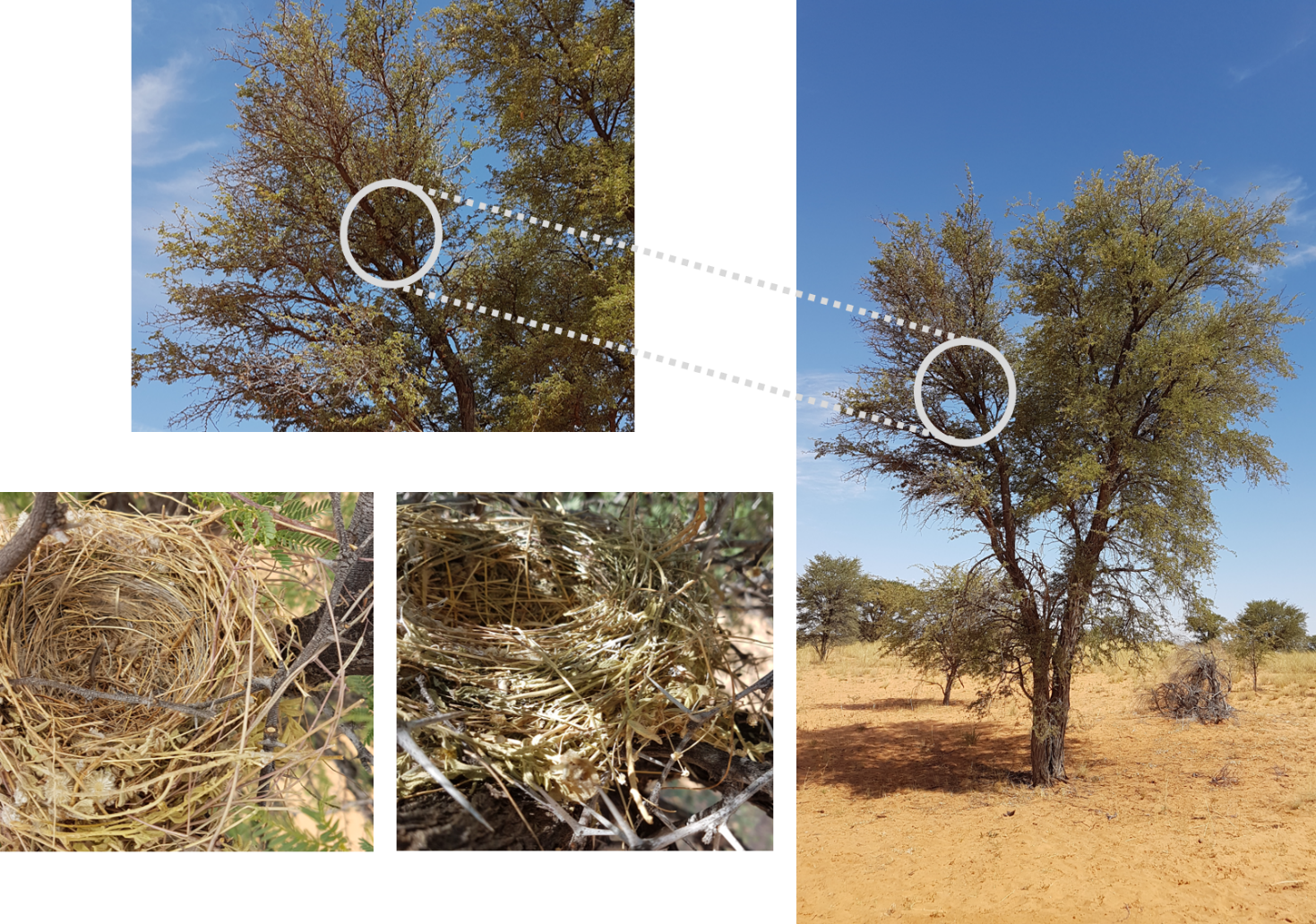


Figure 2: Southern Pied Babblers *Turdoides bicolor* build open cup nests, usually in camelthorn *Vachellia erioloba* trees. © Amanda R. Bourne

# Results

Example 1: The dominant female of a group consisting of three adult pied babblers incubated for four straight hours (12h12 – 16h17) during the hottest part of the day on a day where the daily maximum air temperature was 40.2˚C [pied babblers exhibit compromised foraging efficiency and an resultant inability to maintain body mass overnight at temperatures > 35.5˚C (du Plessis, Martin, Hockey, Cunningham, & Ridley, 2012)]. She panted 88.1% of the time during that particular incubation period [she was clearly visible in the nest from an observation point 10-15m away; panting is an evaporative cooling behaviour used to maintain body temperature in a variety of mammals and birds (Schmidt-Nielson, 1990)]. When she left the nest at 16h17, she exhibited signs of severe heat stress and dehydration, including loss of coordination and diarrhoea (McKechnie et al., 2017). She was not replaced by another group member at this time and left the nest unattended for ~2 hours, returning shortly after sunset, at 18h51, to incubate overnight. Soon after alighting from the nest she began to move towards the nearest water source, a livestock trough ~300m away, taking more than an hour to travel the required distance. She had lost 3.9g by the following day, and DLW analysis showed that she had a negative water balance (0.874).

Example 2: At another group of pied babblers (group size = 7), we observed milder signs of dehydration [sluggishness, sunken eyes (Sharpe, Cale, & Gardner, 2019)] in the dominant female after she had incubated for almost 4 hours in the afternoon (13h45-17h30) on a hot day (40.4˚C). When she left the nest, she was not replaced by another group member. She sat motionless in the shade for ~ 1 hour before returning to the nest after sunset to incubate overnight. This group does not have access to standing water in their territory and the bird was not observed drinking after leaving the nest. She had lost 1.4g by the following day, and DLW analysis showed that she had a negative water balance (0.908).

Example 3: At a third group of pied babblers (group size = 6 adults), we recorded unusual incubation behaviour followed by nest abandonment during a breeding attempt that took place during a heat wave. Both male and female dominant individuals were observed flying to drink water immediately after completing incubation bouts, and the pair left the nest unattended for extended periods of time – up to 4 hours. They did not replace each other immediately after completing incubation, as is usual for pied babblers, but only after the nest had been left unattended for at least 40 minutes. The dominant female did most of the incubating compared to the dominant male (6.9 hours vs. 0.9 hours). Daily maximum air temperatures on nine of the eleven days between initiation of incubation and failure of the breeding attempt exceeded 35.5˚C (the average maximum temperature for the incubation period was 37.1˚C). The nest was abandoned after five consecutive days > 35.5˚C, evidenced by the fact that we found two unhatched eggs in the nest after the group had ceased incubation. The hottest day of the heat wave (40.6˚C) occurred the day before the nest was abandoned. The dominant female had lost 1.9g and the dominant male had lost 3.4g by the following day. Water balance was not measured in either bird on that day.

# Discussion and Conclusions

Without detailed physiological work we cannot be sure whether the behavioural and other symptoms we recorded do in fact signify dehydration, or at least water stress, in incubating pied babblers. Nonetheless, our observations of extended incubation recesses and signs of apparent dehydration in several dominant birds after they had incubated for long periods of time on hot afternoons suggest that 1) incubation for long periods of time on hot afternoons carries a high water cost for the incubating bird; 2) incubating birds will continue to incubate their clutches for as long as possible at high temperatures, until they reach a perceived physiological limit; and 3) once that limit is reached, incubating birds will vacate the nest, whether there is another group member to replace them or not, in order to seek shade, rest, and/or water, thereby avoiding their own lethal dehydration. Considering that we found one clutch that was definitely abandoned, reduced nest attendance on hot afternoons may suggest progress towards eventual nest abandonment (Clauser & McRae, 2017; Sharpe et al., 2019; Stoleson & Beissinger, 1999) as a result of unacceptable dehydration risk. Future research could usefully explore the relationships among temperature, incubation effort, thermoregulatory behaviour, and hydration status in birds breeding in hot and dry environments.

# Acknowledgements

We thank the management teams at the Kuruman River Reserve (KRR) and surrounding farms, Van Zylsrus, South Africa, for making the work possible. The KRR was financed by the Universities of Cambridge and Zurich, the MAVA Foundation, and the European Research Council (Grant No. 294494 to Tim Clutton-Brock), and received logistical support from the Mammal Research Institute of the University of Pretoria. We thank Sello Matjee, Paige Ezzey, and Lesedi Moagi for fieldwork assistance during 2016–2019. This work was funded by the DST-NRF Centre of Excellence at the FitzPatrick Institute for African Ornithology, the University of Cape Town, the Oppenheimer Memorial Trust (Grant No. 20747/01 to ARB), the British Ornithologists’ Union, the Australian Research Council (Grant No. FT110100188 to ARR), and the National Research Foundation of South Africa (Grant No. 99050 to SJC). The opinions, findings and conclusions are those of the authors alone, and the National Research Foundation accepts no liability whatsoever in this regard.

# References

Bourne, A. R., Mckechnie, A. E., Cunningham, S. J., Ridley, A. R., Woodborne, S. M., & Karasov, W. H. (2019). Non‐invasive measurement of metabolic rates in wild, free‐living birds using doubly labelled water. *Functional Ecology*, *33*(1), 162–174.

Bueno-Enciso, J., Barrientos, R., Ferrer, E. S., & Sanz, J. J. (2017). Do extended incubation recesses carry fitness costs in two cavity-nesting birds? *Journal of Field Ornithology*, *88*(2), 146–155. doi:10.1111/jofo.12194

Clauser, A. J., & McRae, S. B. (2017). Plasticity in incubation behaviour and shading by king rails (*Rallus elegans*) in response to temperature. *Journal of Avian Biology*, *48*(4), 479–488. doi:10.1111/jav.01056

Conrey, R. Y., Skagen, S. K., Yackel Adams, A. A., & Panjabi, A. O. (2016). Extremes of heat, drought and precipitation depress reproductive performance in shortgrass prairie passerines. *Ibis*, *158*(3), 614–629. doi:10.1111/ibi.12373

du Plessis, K. L., Martin, R. O., Hockey, P. A. R., Cunningham, S. J., & Ridley, A. R. (2012). The costs of keeping cool in a warming world: Implications of high temperatures for foraging, thermoregulation and body condition of an arid-zone bird. *Global Change Biology*, *18*(10), 3063–3070.

DuRant, S. E., Willson, J. D., & Carroll, R. B. (2019). Parental effects and climate change: will avian incubation behavior shield embryos from increasing environmental temperatures? *Integrative and Comparative Biology*, *59*(4), 1068–1080. doi:10.1093/icb/icz083

Hemmings, N., & Birkhead, T. R. (2015). *Consistency of passerine embryo development and the use of embryonic staging in studies of hatching failure*.

McKechnie, A. E. (2019). Physiological and morphological effects of climate change. In P. O. Dunn & A. P. Moller (Eds.), *Effects of Climate Change on Birds* (pp. 120–133). Oxford: Oxford University Press. Retrieved from https://books.google.co.za/books?hl=en&lr=&id=B5ygDwAAQBAJ&oi=fnd&pg=PA120&ots=yOqTbX-lcW&sig=PmHoz6or3v7M_eID_I40yKhEhFs#v=onepage&q&f=false

McKechnie, A. E., Gerson, A. R., McWhorter, T. J., Smith, E. K., Talbot, W. A., & Wolf, B. O. (2017). Avian thermoregulation in the heat: evaporative cooling in five Australian passerines reveals within-order biogeographic variation in heat tolerance. *The Journal of Experimental Biology*, *220*(Pt 13), 2436–2444. doi:10.1242/jeb.155507

Nagy, K. A. (1980). CO2 production in animals: analysis of potential errors in the doubly labeled water method. *The American Journal of Physiology*, *238*(5), R466-73.

Nelson-Flower, M. J., Hockey, P. A. R., O’Ryan, C., Raihani, N. J., Du Plessis, M. A., & Ridley, A. R. (2011). Monogamous dominant pairs monopolize reproduction in the cooperatively breeding pied babbler. *Behavioral Ecology*, *22*(3), 559–565.

O’Connor, R. S., Brigham, R. M., & McKechnie, A. E. (2018). Extreme operative temperatures in exposed microsites used by roosting rufous-cheeked nightjar (Caprimulgus rufigena): implications for water balance under current and future climate conditions. *Canadian Journal of Zoology*.

Ridley, A. R. (2016). Southern pied babblers: The dynamics of conflict and cooperation in a group-living society. In J. L. Dickinson & W. Koenig (Eds.), *Cooperative Breeding in Vertebrates: Studies of Ecology, Evolution, and Behavior* (pp. 115–132). Cambridge: Cambridge University Press.

Ridley, A. R., & Raihani, N. J. (2007). Variable postfledging care in a cooperative bird: causes and consequences. *Behavioral Ecology*, *18*(6), 994–1000.

Ridley, A. R., & Thompson, A. M. (2011). Heterospecific egg destruction by Wattled Starlings and the impact on Pied Babbler reproductive success. *Ostrich*, *82*(3), 201–205. doi:10.2989/00306525.2011.618247

Ridley, A. R., & van den Heuvel, I. (2012). Is there a difference in reproductive performance between cooperative and non-cooperative species? A southern African comparison. *Behaviour*, *8*, 821–848.

Schmidt-Nielson, K. (1990). *Animal physiology: adaptation and environment.* Cambridge, UK: Cambridge University Press.

Sharpe, L., Cale, B., & Gardner, J. L. (2019). Weighing the cost: the impact of serial heatwaves on body mass in a small Australian passerine. *Journal of Avian Biology*, jav.02355. doi:10.1111/jav.02355

Stoleson, S. H., & Beissinger, S. R. (1999). Egg viability as a constraint on hatching synchrony at high ambient temperatures. *Journal of Animal Ecology*, *68*, 951–962.

Wada, H., Kriengwatana, B., Allen, N., Schmidt, K. L., Soma, K. K., & MacDougall-Shackleton, S. A. (2015). Transient and permanent effects of suboptimal incubation temperatures on growth, metabolic rate, immune function and adrenocortical responses in zebra finches. *Journal of Experimental Biology*. doi:10.1242/jeb.114108

Wiley, E. M., & Ridley, A. R. (2018). The benefits of pair bond tenure in the cooperatively breeding pied babbler (*Turdoides bicolor*). *Ecology and Evolution*, *8*(14), 7178–7185.
